# Supplementary material for: Understanding how community antiretroviral delivery influences engagement in HIV care: a qualitative assessment of the Centralised Chronic Medication Dispensing and Distribution programme in South Africa
Source: BMJ Open. 2020 May 15;10(5):e035412. doi: 10.1136/bmjopen-2019-035412 (PMC7245406; doi:10.1136/bmjopen-2019-035412)
Supplement: Supplementary data [file bmjopen-2019-035412supp001.pdf]

# Understanding how community antiretroviral delivery programmes influence engagement in HIV care: a qualitative assessment of the Centralised Chronic Medication Dispensing and Distribution programme in South Africa

## Supporting Information

**Table of Contents** **Page**


1. Participant in-depth interview topic guide 1
2. Staff in-depth interview topic guide 3
3. Participant focus group topic guide – CCMDD 5

**Patient and service provider perspectives regarding point of care viral load testing and differentiated care: an ancillary study to the STREAM study**

**Participant in Depth Interview Topic Guide**

The overarching objectives of this interview topic guide are to:

- Understand participants experience of laboratory and point of care viral load testing
- Understand participants experience of receiving care from an enrolled nurse compared to a professional nurse
- Understand participants experience of receiving HIV care at clinics, community based organisations and pharmacies.

The interview should last approximately 30 to 60 minutes. The questions are indicative of the topics covered and will be shaped by participants' discussions and concerns. The interview will be conducted by a skilled Zulu facilitator and recorded without participant's name or personal details, but with their consent and permission for further analysis.

## Introduction and background experience of HIV care

Tell me a little about yourself, your age, where you stay, what you do to get by, and where you have been getting your treatment from.

## Laboratory and point of care testing

Tell me about the last time you had a blood test as part of your HIV care. When did you get the results?

How did you feel while you were waiting for the results?

Tell me about the results that you were given. How did you feel about these results?

If you had point of care blood taken, what were the good things and what were the bad things about this approach?

What would you want us to do differently with blood testing? Why do you think this would help?

## Antiretroviral therapy collection at community centres and pharmacies

Tell me about the last time that you collected your antiretrovirals (ARVs). Where did you go, who did you talk to and how was the experience?

What were the good things about collecting treatment there, and what were the bad things? If you ever collected treatment at a community centre or community pharmacy, can you tell me the story of the first time you went there?

What other things are going on in your life which make it easier or harder to collect your treatment?

## Enrolled nurse and professional nurse care

Can you tell me about a time when you felt unwell and told your problem to a nurse? What happened? How did you feel about the care you received?

Tell me about the last time you came to clinic and saw a nurse. What did the nurse do for you that day?

2

If you saw an enrolled nurse, rather than a professional nurse, how did you feel about the care you received from the enrolled nurse?

What were the good and bad things about seeing an enrolled nurse?

**Wrap Up**

Have you got any other ideas that may help improve HIV care services?



Dorward J, *et al. BMJ Open* 2020; 10:e035412. doi: 10.1136/bmjopen-2019-035412

**Patient and service provider perspectives regarding point of care viral load testing and differentiated care: an ancillary study to the STREAM study**

**Staff in Depth Interview Topic Guide**

The overarching objectives of this interview are to:

- Understand staff experience and views on laboratory and point of care viral load testing
- Understand staff experience and views regarding care provision by an enrolled nurse compared to a professional nurse
- Understand staff experience and views regarding care provision at clinics, community based organisations and pharmacies.

The interview should last approximately 30 to 60 minutes. The questions are indicative of the topics covered and will be shaped by the discussions and interviewees concerns. The interview will be conducted by a skilled facilitator and recorded without participant's name or personal details, but with their consent and permission for further analysis.

## Introduction and background experience of HIV care

Tell me a little about yourself, where you have worked in the past, where you work now and what your main role at work is.

## Laboratory and point of care testing

Tell me about any point of care (POC) tests that are used in your clinic/area of work, and your experience of using them.

What do you like and not like about each of these POC tests?

How could these POC tests be improved?

In your experience, what are the most important features of POC tests?

Tell me about the last time you told a patient that they had a high viral load. If this was a POC test, do you think giving the result on the day made any difference? What difference would there have been if it was a laboratory test?

If this was a laboratory test, what difference would there have been if it was a POC test?

Tell me the ways that you think POC testing could improve HIV care?

## ART collection at community centres and pharmacies (CCMDD)

Tell me about your experience of the Centralised Chronic Medicines Dispensing and Distribution (CCMDD) system. What are the good things and bad things about it?

Tell me the main reasons that patients like CCMDD.

Tell me the main reasons that patients <u>don't</u> like CCMDD.

How do you think the CCMDD system could be improved?



Dorward J, *et al. BMJ Open* 2020; 10:e035412. doi: 10.1136/bmjopen-2019-035412

4

## Enrolled nurse and professional nurse care

What do you feel are the most important roles that enrolled nurses currently perform in the HIV programme?

What do you feel are the most important roles that professional nurses currently perform in the HIV programme?

Do you think any professional nurse roles could be done by enrolled nurses? Which ones and why/why not?

Are there any roles that professional nurses DON'T currently perform that you think they could or should do?

In our study, enrolled nurses have been seeing stable patients. What advantages and disadvantages do you think this approach has?

Are there any ways that you think we could improve this approach?

## Wrap Up

Have you got any other ideas of how POC testing, community ART delivery/CCMDD or using enrolled nurses in innovative ways may help improve HIV care services?



Dorward J, *et al. BMJ Open* 2020; 10:e035412. doi: 10.1136/bmjopen-2019-035412

**Patient and service provider perspectives regarding point of care viral load testing and differentiated care: an ancillary study to the STREAM study**

**Participant Focus Group Topic Guide - CCMDD**

We will arrange to meet participants enrolled in the study in a private location at or near the clinic to participate in a focus group. Between 5-10 participants will be in each focus-group.

The overarching objectives of this focus group topic guide are to:

- Understand participants experience of receiving HIV care at clinics, community based organisations and pharmacies.

The focus groups should last approximately 45-75 minutes. The questions are indicative of the topics covered and will be shaped by participants' discussions and concerns. The group will be facilitated by a skilled Zulu facilitator and recorded without participants' names or personal details, but with their consent and permission for further analysis

## Introduction and background experience of HIV care

Tell us a little about your lives, your age, where you stay, what you do to get by, and where you have been getting your treatment from.

## ART collection at community centres and pharmacies

Can someone who had a problem getting their treatment in the last few months tell us the story about what happened?

Did anyone else in the group experience something similar and manage to find a way around it – can you describe this please?

Is there anything that the CAPRISA or CDC clinic could have done to help you overcome these problems?

Does anyone have examples of other strategies that you or other people use to fit collecting medication into your everyday life?

Some people have been able to collect their treatment from community pharmacies and community centres. Can someone who did this tell us about the first time they went to collect their treatment there, where did you go, who did you talk to and how was the experience?

Does anyone else have a similar story? What were the good things about collecting treatment there, and what were the bad things? How did you feel when staff first told you that you could collect treatment at the community centre or pharmacy? Can anyone tell us about what happens when they come back to the clinic for review every 6 months?

## Wrap Up

Have any of you got any other ideas that may help improve HIV care services?
