# Supplementary material for: Understanding how community antiretroviral delivery influences engagement in HIV care: a qualitative assessment of the Centralised Chronic Medication Dispensing and Distribution programme in South Africa
Source: BMJ Open. 2020 May 15;10(5):e035412. doi: 10.1136/bmjopen-2019-035412 (PMC7245406; doi:10.1136/bmjopen-2019-035412)
Supplement: Supplementary data [file bmjopen-2019-035412supp002.pdf]

Table S1: Coding frame based on Framework of Social Practice

Code	Description	Quote
**MATERIALITIES:** Physical 'things' that impact on engagement in HIV services		
**Characteristics of HIV services**	The physical aspects of HIV services that determine access	
Waiting times	Descriptions of waiting times in HIV services	*"I think collecting at [the private pharmacy] is quicker than here, when I came back here at the clinic I waited for a good two hours."* Focus group 1, Participant 7, Male<br><br>*"They [patients] like CCMDD, [because] when they go to the pick-up points there's no queues like here in the clinic. They come in, they go straight to the pharmacies then they get their pack."* Staff interview 4*
Numbers of patients	Descriptions of the number of patients receiving treatment	*"They [nurses] are always in a rush and there are a lot of us. Maybe we are more than 100 and there's a queue."* Focus group 1, Participant 4, Female<br><br>Interviewer: *"Ok, so the first time you went to the hall [CCMDD community pick-up point], how was it?"* Participant: *"It was nice because there was not a lot of us, I would just take my ID out and then take my treatment and leave."* Focus group 2, Participant 2, Male
Staffing levels	Descriptions of staffing levels in HIV services	*"There is not enough staff [in the clinic], everyone is rushing to finish... Maybe only three nurses are working, and they are seeing all these people. You are also rushing and she is also rushing and you are thinking as a patient that you left a lot of people outside [waiting to be seen after you]. So adding more staff would help."* Client interview 3, Male

Dorward J, *et al*. *BMJ Open* 2020; 10:e035412. doi: 10.1136/bmjopen-2019-035412

Code	Description	Quote
Flexibility of HIV services	Descriptions of (in)flexibility of HIV services with regard opening hours and ability to change appointment days	*"Collecting on weekends would be a great idea, because you will not have an excuse especially if you are not working. Even at work they will have a problem if you have a specific day where you don't come to work or leave early [to collect ART]."* Focus group 2, Participant 1, Male<br>*"Collecting… [in CCMDD] saves a lot of time, you don't spend 5 minutes. It doesn't get full, you can come anytime if it's still open. You just get in there take your medication and leave."* Focus group 3, Participant 5, Female
Accessing other medical care apart from ART	Descriptions of how the ability to access other, non-ART related care influenced engagement with HIV services	*"It's quicker to just collect [ART] because you know how you are taking your treatment… When all these things [measuring vitals] are done you will be delayed."* Focus group 2, Participant 8, Female<br>*"The problem with it [CCMDD] is that you only come to collect your medication and then leave, they don't do other things as well. Like checking your blood pressure and other things to know how your health is."* Client interview 5, Female
Maintaining confidentiality	Descriptions of how the design of the service impacts on confidentiality	*"But at the nearby clinics, most of the nurses just display everything [ART] in front of everyone… there is no privacy in these clinics."* Focus group 1, Participant 8, Female<br>*"When your neighbours see you sitting there they will start to ask you questions. Even when they don't know why you are there, but they will know at the end why."* Focus group 1, Participant 6, Male
ART not being available in facility or pickup point	Descriptions of attending services but ART not being available	*"Sometimes you find that there is no treatment [in the clinic] and people would be scheduled for another date when the treatment is available."* Client interview 21, Male

Code	Description	Quote
Distance to HIV services	Descriptions of the distance and travel to HIV services affecting engagement in care	*"There are people who live far [from the clinic]… you find that they don't even have money for transport."* Client interview 6, Female
Barriers to engagement with CCMDD	Descriptions of factors that stop people from being referred into CCMDD or continuing to collect there (see Table 3)	See Table 3 for specific quotes
**Poverty**	The dynamics of lacking financial and material resources (e.g. food, other medication to manage side effects) that would support engagement with HIV services.	*"Fetching medication is problematic. You find that you don't have money, you need to fetch your medication and you don't have it [money]."* Client interview 1, Female
**COMPETENCIES:** Knowledge and skills that impact on engagement in HIV services		
**Communication between healthcare workers and patients**	The role of healthcare workers in communicating competencies and also clinical information about HIV and treatment to patients	*"Patient education… should be constant, it should be regular, regular talking with the patients, teaching patients how to live positively, considering things like the use of condoms, proper dieting and… the function of adherence."* Staff interview 4<br><br>Interviewer: *"When you ask the nurse about your results, what do they say?"*<br>Participant: *"You even get afraid to ask, because they don't have time, they are always rushing to see the next person."* Focus group 3, Participant 4, Female
Healthcare worker attitude	Descriptions of the importance of healthcare worker attitude in imparting these competencies towards patients	*"They [healthcare workers] must not judge us, they must do their job and what they were employed to do. Also, they need to learn to communicate, nurses can't communicate with people. You make one small mistake [and] they shout at you and talk to you the way they like."* Client interview 19, Female

Code	Description	Quote
**To manage stigma**	Knowledge and skills needed to manage the (non)disclosure of one's HIV status to others, and to deal with stigma from others including partners, family members and the wider community.	*"I didn't want to go to the clinic by where I live because I was afraid of people's eyes. Because when you start this [ART] people look at you in different way."* Client interview 20, Male
**To negotiate problems with HIV services**	Descriptions of strategies and skills that people have developed to mitigate problems with HIV services	*"You see, I make it a point that when it's my appointment date I wake up very early to get to the clinic… I will be here for the whole day… because there are a lot of people… You must wait that long and sometimes you get so tired and you wish you can just leave. What can we say? We must wait because it's our lives."* Client interview 12, Female<br>*"If you don't have a day-off on that day you can send someone to collect the medication for you."* Client interview 1, Female
**Social support**	The use of family and or the wider community as a source of knowledge to help maintain engagement with HIV services	"*[After starting ART] I did have a bad dream on the first day... But after that there were no problems. I asked other people, and they told me that they did have side-effects on the first day, you will have bad dreams and I didn't have a problem with that, it passed. Because when I asked other people they said, yes, it happens. But the ARVs are no longer the same as the previous ones, these ones are better because you only take one pill at night. Yes, I continued I took the medication."* Client interview 1, Female
**Maintaining a healthy lifestyle**	Knowledge and skills needed to maintain recommended lifestyle changes for people living with HIV	*"They were advising us that we shouldn't tell ourselves that we are going to die you see… that if you are person living with HIV you practice safe sex and not sleep around and try to eat healthy things in life. If you smoke reduce a bit and [reduce] drinking. General things were being spoken that we need to deal with."* Client interview 10, Male

Dorward J, *et al. BMJ Open* 2020; 10:e035412. doi: 10.1136/bmjopen-2019-035412

Code	Description	Quote
**MEANINGS:** The meanings that are attached to HIV and engagement with HIV services		
**Stigma**	Prejudice and discrimination experienced by people living with HIV. Can be internal ('self-stigma') or external (enacted by others) and both perceived or actual.	*"[if there is a separate queue for ART] it's easier to be discriminated against, because they know what you are here for, so it's better for a person not to know what you are queuing for."* Focus group 1, Participant 4, Female<br>*"I wish they could make this disease [HIV] like flu, and not something scary. That is the reason why people don't even want to come close to you."* Focus group 1, Participant 7, Male<br>*"My concern is the stigma we have as infected people, we get treated as people who don't live in this world. So, we try to hide ourselves."* Focus group 2, Participant 4, Male
**Normalisation of HIV**	The portrayal of HIV as a common and normal experience share with many others, that does not have to affect health if ART is taken.	Interviewer: *"When you found out [that you were HIV-positive] … how did you feel about that?"*<br>Participant: *"I didn't feel like a different person, I didn't see any difference, I saw myself as a normal person who needs to take care of herself and take her medication properly so life can go on. I didn't feel any change and I wasn't sick at all."* Client interview 5, Female
**Meanings of CCMDD**	Descriptions of the meaning associated with being referred to CCMDD including reasons for being referred	*"They transferred me to CCMDD because I was taking my treatment properly and I also know that I was taking my treatment well."* Focus group 2, Participant 7, Male
**Public and private treatment**	The meanings associated with receiving government funded versus private care	*"Sometimes you wait for other [customers] to buy and they [private pharmacy staff] will serve the other people before you. Even if you are in the queue, it's like they are sifting us [public CCMDD clients] from the crowd."* Focus group 1, Participant 5, Female

Dorward J, *et al*. *BMJ Open* 2020; 10:e035412. doi: 10.1136/bmjopen-2019-035412

Code	Description	Quote
		"They [private pharmacy staff] were paying attention to the customers who were coming to buy. Because we don't come with money, we only come with our [CCMDD] cards and ID's [so] they were not paying attention to us." Client interview 8, Female
**Experience of HIV and treatment**	Having had personal experience, or having witnessed in others, the effects of HIV and life-saving treatment. Having faith in treatment and HIV services in restoring health and life	*"As for me, even when I walk on the streets I feel healthy and confident because I know I am taking my medication properly."* Focus group 2, Participant 9, Female
**OTHER LIFE PRACTICES:** Other repeated activities of daily life that clients engage in that impact on engagement in HIV services		
**Employment**	The duties and daily activities of employment and formal and informal work	*"I even go after work, I knock off at 4 and go there [CCMDD pick-up point] and take my medication. Even during my break time, I go, or when I am swamped I ask [my supervisor] and quickly rush there take my medication and come back… What I love [about CCMDD] is that I don't need to take a day off at work to collect medication at the clinic and wait for the whole day. Now at work I ask for that 20 minutes and go there [to CCMDD]. It's fine. I don't skip work, and there is no one that sees me."* Client interview 12, Female<br><br>*"I think it [CCMDD] is good, because clinics get full. It's better because if you work you can quickly go there [the community pick-up point], collect and come back. I think it's a good method."* Client interview 4, Female
**Family life**	Activities of daily life that revolve around family	*"Last time [I had to collect ART from the clinic] you know what happened? I had to send my wife. I'd got a part time job, so I couldn't manage to come. [So I asked her], 'Please just go with my card to collect my medication'. She doesn't want to come back here [to the clinic] ever again.*

Code	Description	Quote
		Because of the time that she spent [waiting for treatment].” Client interview 14, Male

*To protect participant confidentiality, we have not provided complete demographic details of healthcare worker for individual quotes.

Dorward J, *et al. BMJ Open* 2020; 10:e035412. doi: 10.1136/bmjopen-2019-035412
