# Supplementary material for: Understanding how community antiretroviral delivery influences engagement in HIV care: a qualitative assessment of the Centralised Chronic Medication Dispensing and Distribution programme in South Africa
Source: BMJ Open. 2020 May 15;10(5):e035412. doi: 10.1136/bmjopen-2019-035412 (PMC7245406; doi:10.1136/bmjopen-2019-035412)
Supplement: Author's manuscript [file bmjopen-2019-035412.draft_revisions.pdf]

BMJ Open is committed to open peer review. As part of this commitment we make the peer review history of every article we publish publicly available.

When an article is published we post the peer reviewers' comments and the authors' responses online. We also post the versions of the paper that were used during peer review. These are the versions that the peer review comments apply to.

The versions of the paper that follow are the versions that were submitted during the peer review process. They are not the versions of record or the final published versions. They should not be cited or distributed as the published version of this manuscript.

BMJ Open is an open access journal and the full, final, typeset and author-corrected version of record of the manuscript is available on our site with no access controls, subscription charges or pay-per-view fees (http://bmjopen.bmj.com).

If you have any questions on BMJ Open's open peer review process please email info.bmjopen@bmj.com

BMJ Open

Understanding how community antiretroviral delivery programmes influence engagement in HIV care: a qualitative assessment of the Centralised Chronic Medication Dispensing and Distribution programme in South Africa

Journal:	*BMJ Open*
Manuscript ID	bmjopen-2019-035412
Article Type:	Original research
Date Submitted by the Author:	30-Oct-2019

Complete List of Authors:	Dorward, Jienchi; University of Oxford, Nuffield Department of Primary Care Health Sciences; Centre for the Aids Programme of Research in South Africa,<br>Msimango, Lindani; Centre for the Aids Programme of Research in South Africa,<br>Gibbs, Andrew; South African Medical Research Council, Gender and Health Research Unit; University of KwaZulu-Natal, Centre for Rural Health<br>Shozi, Hlengiwe; Centre for the Aids Programme of Research in South Africa,<br>Tonkin-Crine, Sarah ; University of Oxford, Nuffield Department of Primary Care Health Sciences; University of Oxford, 5 NIHR Health Protection Research Unit in Healthcare Associated Infections and Antimicrobial Resistance<br>Hayward, Gail; University of Oxford, Nuffield Department of Primary Care Health Sciences<br>Butler, C; University of Oxford, Nuffield Department of Primary Care Health Sciences<br>Ngobese, Hope; Ethekwini Municipality, Health Unit<br>Drain, Paul; University of Washington, Departments of Global Health, Medicine and Epidemiology<br>Garrett, Nigel; Centre for the Aids Programme of Research in South Africa, ; University of KwaZulu-Natal, Discipline of Public Health Medicine, School of Nursing and Public Health,

Keywords:	Public health < INFECTIOUS DISEASES, International health services < HEALTH SERVICES ADMINISTRATION & MANAGEMENT, HIV & AIDS < INFECTIOUS DISEASES, PRIMARY CARE

SCHOLARONE™
Manuscripts

I, the Submitting Author has the right to grant and does grant on behalf of all authors of the Work (as defined in the below author licence), an exclusive licence and/or a non-exclusive licence for contributions from authors who are: i) UK Crown employees; ii) where BMJ has agreed a CC-BY licence shall apply, and/or iii) in accordance with the terms applicable for US Federal Government officers or employees acting as part of their official duties; on a worldwide, perpetual, irrevocable, royalty-free basis to BMJ Publishing Group Ltd ("BMJ") its licensees and where the relevant Journal is co-owned by BMJ to the co-owners of the Journal, to publish the Work in this journal and any other BMJ products and to exploit all rights, as set out in our licence.

The Submitting Author accepts and understands that any supply made under these terms is made by BMJ to the Submitting Author unless you are acting as an employee on behalf of your employer or a postgraduate student of an affiliated institution which is paying any applicable article publishing charge ("APC") for Open Access articles. Where the Submitting Author wishes to make the Work available on an Open Access basis (and intends to pay the relevant APC), the terms of reuse of such Open Access shall be governed by a Creative Commons licence – details of these licences and which Creative Commons licence will apply to this Work are set out in our licence referred to above.

Other than as permitted in any relevant BMJ Author's Self Archiving Policies, I confirm this Work has not been accepted for publication elsewhere, is not being considered for publication elsewhere and does not duplicate material already published. I confirm all authors consent to publication of this Work and authorise the granting of this licence.

For peer review only

TITLE: UNDERSTANDING HOW COMMUNITY ANTIRETROVIRAL DELIVERY PROGRAMMES INFLUENCE ENGAGEMENT IN HIV CARE: A QUALITATIVE ASSESSMENT OF THE CENTRALISED CHRONIC MEDICATION DISPENSING AND DISTRIBUTION PROGRAMME IN SOUTH AFRICA

AUTHORS

Jienchi Dorward[1,2§], Lindani Msimango[1], Andrew Gibbs[3,4], Hlengiwe Shozi[1], Sarah Tonkin-Crine[2,5], Gail Hayward[2], Chris Butler[2], Hope Ngobese[6], Paul K Drain[7,8,9], Nigel Garrett[1,10]

AFFILIATIONS

1. Centre for the AIDS Programme of Research in South Africa (CAPRISA), University of KwaZulu–Natal, Durban, South Africa.
2. Nuffield Department of Primary Care Health Sciences, University of Oxford, Oxford, UK.
3. Gender and Health Research Unit, South African Medical Research Council, Pretoria, South Africa.
4. Centre for Rural Health, School of Nursing and Public Health, University of KwaZulu-Natal, Durban, South Africa.
5. NIHR Health Protection Research Unit in Healthcare Associated Infections and Antimicrobial Resistance, University of Oxford, Oxford, UK
6. Ethekwini Municipal Health Department, Durban, South Africa
7. Department of Global Health, Schools of Medicine and Public Health, University of Washington, Seattle, USA.
8. Department of Medicine, School of Medicine, University of Washington, Seattle, USA.
9. Department of Epidemiology, School of Public Health, University of Washington, Seattle, USA.
10. Discipline of Public Health Medicine, School of Nursing and Public Health, University of KwaZulu-Natal, Durban, South Africa.

[§] Corresponding author: Dr Jienchi Dorward

Nuffield Department of Primary Care Health Sciences, University of Oxford,
Gibson Building 1st Floor, Radcliffe Observatory Quarter, Woodstock Road, Oxford, OX2 6GG
Email: Jienchi.Dorward@phc.ox.ac.uk

E-mail addresses of authors:

JD: jienchi.dorward@phc.ox.ac.uk

LM: lindani.msimango@caprisa.org

AG: Andrew.Gibbs@mrc.ac.za

HS: Hlengiwe.Shozi@caprisa.org

STC: sarah.tonkin-crine@phc.ox.ac.uk

GH: gail.hayward@phc.ox.ac.uk

CB: christopher.butler@phc.ox.ac.uk

HN: hope.ngobese@durban.gov.za

PD: pkdrain@uw.edu

NG: nigel.garrett@caprisa.org

Keywords: differentiated care; community; antiretroviral therapy; HIV; South Africa

Target Journal: BMJ Global Health

Word counts: Abstract 300/300. Main text: 4381/5000

For peer review only

ABSTRACT (300/300)

Introduction

Providing antiretroviral therapy (ART) for the 37.9 million people living with HIV globally requires efficient, client-centred models of ART delivery. In South Africa, the Centralised Chronic Medication Dispensing and Distribution (CCMDD) programme allows over 1 million people to collect ART from community pick-up points, instead of attending clinics. We aimed to explore how CCMDD influences engagement in HIV care.

Methods

We performed in-depth interviews and focus group discussions with clients on ART and health-care workers in Durban, South Africa. We analysed transcripts using deductive thematic analysis, with a framework informed by ‘theories of practice’.

Results

Between March and August 2018 we undertook 25 interviews and four focus groups with a total of 55 clients, and interviewed eight healthcare workers. Using ‘theories of practice’, we found that CCMDD overcame material barriers to attending clinics, changed the meanings associated with collecting ART and was less disruptive to other social practices in clients’ lives. In clinics, long waiting times, poor confidentiality, and restricted opening hours discouraged clients from engagement with HIV care. These issues hindered education from healthcare workers, and conflicted with client’s employment. In contrast, CCMDD allowed quicker and more convenient ART collection at private pharmacies and community pick-up points, reducing disruption to clients’ lives. This particularly benefitted some employed clients, helped to normalise HIV, and was seen as a reward by clients who were taking ART well. However, at private pharmacies some clients reported receiving inferior treatment compared to paying customers, and some worried about inadvertently revealing their HIV status. Lastly, clients and healthcare workers had to negotiate problems with implementation of CCMDD.

Conclusions

Clients and healthcare workers reported several benefits of this community ART delivery programme, which facilitated engagement in HIV care. Further work is needed to improve CCMDD implementation and assess its effectiveness on ART outcomes and costs.

For peer review only - http://bmjopen.bmj.com/site/about/guidelines.xhtml

ARTICLE SUMMARY

Strengths and limitations of this study

- This is one of the first qualitative assessments of the Centralised Chronic Medication Dispensing and Distribution programme (CCMDD), which provides ART to over 1 million people in South African communities.
- Our analysis is strengthened by the use of 'theories of practice', which highlights the different components of the social practice of engagement in HIV care, and how these interact and may be influenced by CCMDD.
- Participants in our study were part of a clinical trial at a large urban clinic, meaning our findings may not be generalisable to other settings, such as rural areas.
- Lastly, the CCMDD programme is adapting quickly, and some of the early implementation problems that we report may now have been resolved.

BACKGROUND

Expanding access to life-saving antiretroviral therapy (ART) for people with HIV has been one of the biggest global health successes of the 21st century. Since 2004, the number of people on ART globally has increased more than ten-fold to 23.3 million, while HIV related mortality has dropped from 1.7 million annual deaths to 0.8 million.[1] ART is now recommended for all people living with HIV, meaning that a further 14.6 million people still require treatment, mainly in low- and middle-income countries.[1] Therefore, ART programmes in these settings will need to continue expanding, while also providing treatment through efficient, convenient services that encourage life-long engagement in HIV care.

Several new models of 'differentiated ART delivery' have been developed, including providing ART through community adherence clubs (where clients meet in a community group to collect ART), delivering ART to decentralized pick up points, and increasing the intervals between ART collection dates using multi-month prescribing.[2] These services aim to provide streamlined, efficient treatment in a client-centred manner for people who are doing well on ART, thereby allowing more resources to be directed towards initiating people on treatment and to managing those who are less well.[3,4]

South Africa currently runs the largest differentiated ART delivery programme in the world; the Centralized Chronic Medication Dispensing and Distribution Programme (CCMDD). Non-pregnant adults who are stable on ART and have had two consecutive suppressed HIV viral

For peer review only - http://bmjopen.bmj.com/site/about/guidelines.xhtml

loads, at least six months apart, can be referred into CCMDD by their ART clinic. In CCMDD, clients collect their treatment bi-monthly at local pickup points, including private pharmacies and community-based organizations.[5] Every six months, they return to the clinic for review and re-referral into CCMDD. An annual viral load is also performed at the clinic assessing whether they remain virally suppressed. If clients in CCMDD are unwell, they can attend their clinic for medical attention. CCMDD is a public-private partnership; patients are referred by healthcare workers in government sector clinics, while a private company is responsible for centrally dispensing medication and delivering it to community pick-up points.[6] As well as HIV treatment, other chronic medications (e.g. antihypertensives) are supplied through CCMDD, to both people with and without HIV. CCMDD was started in 2016 and currently provides ART to over 1.2 million people (personal communication, Roma Ramphal, CCMDD Co-ordinator for KwaZulu-Natal). However, despite its size, there is little published data evaluating how this programme influences engagement in HIV care.

Several different frameworks and theories have been proposed to evaluate factors influencing engagement in HIV care. Skovdal et al [7] propose a theoretical framework around engagement in HIV care as a social practice (Figure 1). ‘Theories of practice’ conceptualise patterns of behaviour as social practices which depend on *materialities* (e.g. clinic accessibility), *competencies* (e.g. knowing how to access services and/or maintain healthy lifestyle), *meanings* (e.g. HIV-associated stigma, the normalisation of HIV as a common experience), and *other life practices* (e.g. employment).[7] These different components dynamically interact to influence the ‘practice’ of engagement in HIV care. Here, we aim to use this framework to explore engagement in clinic-based HIV services, and to analyse how CCMDD may influence the social practice of engagement in HIV care.

METHODS

Study design and setting

We undertook semi-structured in-depth interviews and focus group discussions with clients, and interviews with healthcare workers, who were part of the Simplified TREAtment and Monitoring (STREAM) study, a clinical trial of point-of-care HIV viral load testing and task shifting to enrolled nurses.[8] We previously reported qualitative findings from this study regarding the acceptability of point-of-care viral load testing and enrolled nurse care[9]. Here, we focus on findings related to CCMDD.

For peer review only - http://bmjopen.bmj.com/site/about/guidelines.xhtml

Eligible participants in STREAM were adults aged ≥18 years who were stable on first-line ART for 6 months after initiation. At enrolment, participants were randomized 1:1 to receive point-of-care viral load testing and potential task shifting to enrolled nurses, versus standard laboratory viral load testing and professional nurse care. After six months in the study (one year on ART) participants in both arms who had a suppressed viral load and met other eligibility criteria (clinically stable, non-pregnant, CD4 count >200 cells/mm^3)[5] were offered referral into CCMDD.

Once accepted into CCMDD, participants were able to collect treatment every two months at private pharmacies and community-based organisations. The CCMDD system would automatically send SMS-texts to clients' mobile phones to remind them of their ART collection dates. After six months they were reviewed at the clinic by a nurse. The STREAM study took place at the Prince Cyril Zulu Clinic and the adjacent CAPRISA Ethekwini Clinical Research Site, which are situated next to the main transport hub in central Durban, South Africa. Prince Cyril Zulu Clinic serves a large, urban population and was one of the first clinics to implement CCMDD in Durban, as part of a pilot implementation programme.

For peer review only

Participants

We used a purposive sampling frame to select a range of patients including those who received care in CCMDD, who had suppressed and unsuppressed viral loads, and who received point-of-care testing versus standard laboratory testing in the STREAM trial (Table 1). We approached potential participants in person or by telephone after they had exited the STREAM study, explained reasons for performing the research and obtained informed consent for their participation.

Table 1: Client in-depth interview sampling frame

		**Point-of-care arm**	**Standard-of-care arm**	**Total target**
		Target	Target	
**Viral load result**	**Care provider**			
**< 1000 copies/ml**	CCMDD and/or enrolled nurse	5	5	10
	Professional nurse	5	5	10
**> 1000 copies/ml**	Professional nurse	5	5	10
Total target		15	15	30

CCMDD, Centralised Chronic Medication Dispensing and Distribution programme

For peer review only - http://bmjopen.bmj.com/site/about/guidelines.xhtml

Data collection

Trained research assistants (LM [male], HS [female]) performed interviews and focus groups in IsiZulu or English in a private room in the clinic, using semi-structured topic guides (supplementary material). LM was not previously known to participants. HS was known to patients as she was involved in recruitment and scheduling in STREAM. Neither LM or HS had any involvement in CCMDD or clinical patient care. We audio recorded interviews and focus groups, and took accompanying field notes where appropriate. Recordings and notes were anonymised, transcribed and translated from isiZulu to English where necessary. Participants did not review transcripts or the analysis.

Data analysis and theoretical framework

We performed a deductive thematic analysis[10] using a framework based on predefined themes informed by theories of practice.[7] We identified new themes and codes that were derived from the data and integrated them into the pre-defined themes in the coding frame (Table S1). Data for this analysis was predominantly coded by JD, with assistance by LM, using NVivo 12.0 (QSR International, Melbourne, Australia).

Ethics

Ethical approval for this study was provided by the Biomedical Research Ethics Committee of the University of KwaZulu-Natal and the Institutional Review Board of the University of Washington.

Patient and public involvement

Patients and the public were not involved in the design, recruitment or conduct of the study, beyond being enrolled as participants. Results will be shared with the CAPRISA Community Advisory Board as part of dissemination activities.

RESULTS

Between March and August 2018 we undertook 25 interviews and four focus groups (five to ten participants) with a total of 55 clients, and eight interviews with healthcare workers. One client was both interviewed and also in a focus group. Amongst clients, median age was 31 years, 56.4% were female, 52.7% were employed and 58.2% had collected ART in CCMDD (Table 2). Amongst healthcare workers the median age was 39, 75.0% were women, and all were nurses (Table 2). Interviews lasted 30-60 minutes and focus groups 60-80 minutes. Below, we present

For peer review only - http://bmjopen.bmj.com/site/about/guidelines.xhtml

our findings, with supporting quotes, using two overarching themes of the challenges to engagement in clinic-based services, and the influence of CCMDD on the social practice of engagement in HIV care. Within these themes, we highlight sub-themes based on the four constructs of *materialities*, *competencies*, *meanings* and *other life practices* from theories of practice,[7] which we identified in the analysis (Table S1).

Table 2: Participants demographic and clinical characteristics

Clients			
		Frequency (N=55)	%
Age (years)	Median (IQR)	31 (27 - 37)	
Gender	Male	24	43.6
	Female	31	56.4
Ethnicity	Black African	55	100.0
Educational level	None or primary school	2	3.6
	Did not pass secondary school	14	25.5
	Passed secondary school	27	49.1
	Some tertiary school	12	21.8
Employed	Yes	29	52.7
	No	26	47.3
Income in ZAR per month (approximate USD)	<1000 (70)	23	41.8
	1000-4000 (70-280)	20	36.4
	> 4000 (280)	12	21.8
Has a regular/stable partner	Yes	42	76.4
	No	13	23.6
Number of children	None	10	18.1
	1 or more	45	81.8
Have you disclosed your HIV status to anyone?	Yes	52	94.6
	No	3	5.5
Is your travel time to clinic > 1 hour?	Yes	7	12.7
	No	48	87.2
Distance travelled to clinic ≤ 5 kilometres	Yes	44	80.0
	No	11	20.0
Primary method of travel to clinic	Walking	3	5.5
	Public transport	49	89.1
	Private transport	3	5.5
VL <1000 copies/ml	Yes	49	10.9
	No	6	89.1
Collected ART in CCMDD	Yes	32	58.2
	No	23	41.8

For peer review only

For peer review only - http://bmjopen.bmj.com/site/about/guidelines.xhtml

Healthcare Providers			
Age (years)	Median (IQR)	39 (36 - 42)	
Gender	Male	2	25.0
	Female	6	75.0
Profession	Professional nurse	4	50.0
	Enrolled nurse	4	50.0

IQR, interquartile range

For peer review only

For peer review only - http://bmjopen.bmj.com/site/about/guidelines.xhtml

Challenges to engaging with clinic-based HIV services

Clients and healthcare workers described several ways in which the *materialities* of overcrowded clinic-based HIV services presented challenges to collecting ART. Long queues and restrictive opening times clashed with *other social practices* in clients' lives, such as employment. Rushed clinic appointments undermined clients' ability to learn about their medication and develop *competencies* around taking ART. Furthermore, the physical location and design of clinic services threatened some clients' confidentiality, such that engagement with HIV care could be associated with, or come to *mean*, exposure to HIV-related stigma.

Many clients reported that overcrowded primary care clinics and long waiting times deterred people from collecting ART, and interfered with employment and family responsibilities.

> "*Sometimes I would end up not even collecting them [ART], because of the thought of the queue.*" Focus group 2, Participant 9

> "*I did go to the clinic and the line was massive. So, I couldn't collect [ART], because I had to go back to work.*" Focus group 2, Participant 4

> "*…because the time here [at clinic] clashes with my work time… When you come here [to the clinic] you do it for yourself and when you are at work you do it for the family, you see. You must choose sometimes.*" Client interview 24

Many clients also found that inflexible clinic opening hours (weekdays between 0700 to 1600 hours) clashed with employment opportunities:

> "*Yes, but if I could be able to collect it on weekends… During the week I sometimes have temporary jobs somewhere, so I wish I could collect on weekends.*" Client interview 18

Clients also described how time pressures and rushed appointments prevented communication with healthcare workers, which stopped them from gaining *competency* in understanding and managing their health and HIV treatment.

> *"They would just tell you your bloods are fine and you leave…We don't ask questions, we want to finish and allow other people to come in… there are a lot of patients [waiting] outside who also want to come in and be assisted.*" Client interview 3

> *"Maybe you weren't feeling well two days ago and you want to tell the nurse about it, sometimes you can't… because you are looking at the situation the nurse is under. You*

For peer review only - http://bmjopen.bmj.com/site/about/guidelines.xhtml

see that she has no time for you and she will not tolerate you telling her other things." Focus group 3, Participant 4

Some clients also described being treated badly by clinical staff, further limiting their ability to become *competent* at taking medication.

"Sometimes you speak with [the nurse], and she gives you medication, [but] at the same time she's shouting at you. You can't do anything as a patient, you end up not knowing how you should take the medication." Focus group 3, Participant 4

The physical organization of clinic services sometimes threatened client's confidentiality, potentially revealing their HIV status, which for them *meant* potential exposure to HIV-related stigma.

"There are two polyclinics, there's an old one and the new one… [HIV] positive people go to the 'old poly', so when a person says, "He is an old poly" [it means] they saw you at the 'old poly'. They know when you go to the 'old poly' that you are coming for ART." Focus group 1, Participant 1

Clients developed *competencies* to manage HIV-related stigma and avoid disclosing their HIV status to people that they knew. Some reported pretending to collect treatment for someone else, or choosing to collect their treatment at a clinic far from home.

"[There is] *another thing that makes us collect medication in other areas. We do have clinics in our local communities, [but] we are afraid to collect medication in those clinics because it is not safe… we are in one room with other patients and maybe we are only separated by a wall, so when I enter that door, people will see that I am going to collect my "qo" [ART's]."* Focus group 3, Participant 7

Clients developed other *competencies* to overcome the multiple challenges to engagement in care. These included sending relatives or friends to collect treatment, sharing ART, arriving at clinic very early in the morning to miss queues, and extreme patience because of the life-saving benefits and positive *meanings* associated with receiving ART.

"When it's my appointment date I wake up very early to get to the clinic… I will be here for the whole day… because there are a lot of people… You must wait that long and sometimes you get so tired and you wish you can just leave. What can we say? We must wait because it's our lives." Client interview 12

For peer review only - http://bmjopen.bmj.com/site/about/guidelines.xhtml

The influence of CCMDD on the social practice of engagement in HIV care

Clients and healthcare workers reported that CCMDD introduced changes in the *material circumstances* of ART delivery that had broadly positive impacts on engagement with HIV services.

Many clients and healthcare workers reported that collecting ART in CCMDD was quicker than at the clinic, and with more convenient opening hours.

> *"I think collecting at [the community pharmacy] is quicker than here. When I came back here at the clinic I waited for a good two hours."* Focus group 1, Participant 7

> *"They [patients] like CCMDD, [because] when they go to the pick-up points there's no queues like here in the clinic. They come in, they go straight to the pharmacies then they get their pack."* Staff interview 4

> *"Collecting… [in CCMDD] saves a lot of time, you don't spend 5 minutes. It doesn't get full, you can come anytime if it's still open. You just get in there, take your medication and leave."* Focus group 3, Participant 5

This meant that collecting ART was less disruptive for *other social practices*, such as employment.

> "*Everything [at the CCMDD pick-up point] is perfect and fast. If… I'm supposed to start work at half past eight I can make it, because at eight o'clock I will be there and by ten past or quarter past I'm done.*" Client interview 14

> "*[In CCMDD] the patients… are independent, they can go on a Saturday or after hours to pick up their medication, they don't have to take time off work to come to us [at the clinic].*" Staff interview 5

Several staff felt that CCMDD helped to change the *meanings* associated with HIV treatment by normalizing HIV services.

> *"Being on ART, it disrupted their [clients] normal lives, so with CCMDD people go back to their normal life routine. You don't have to feel that 'I am sick'… you don't have to be burdened with getting treatment.*" Staff interview 1

For peer review only

Specifically, collecting ART in private pharmacies, where clients could be getting non-HIV related medication, helped with maintaining confidentiality.

> *"[In the private pharmacy] they wrap the medication well and pack it well. They don't just give you un-wrapped medication where everyone can see. They wrap them and put them in a plastic bag…it looks like any medication you buy from [the pharmacy]."* Client interview 12

However, stigma still affected the *meanings* of engagement with CCMDD. Some clients who collected from private pharmacies felt that they were treated differently, because they were not paying customers.

> *"They [the private pharmacy staff] were paying attention to the customers who were coming to buy. Because we don't come with money, we only come with our [CCMDD] cards, they weren't paying attention to us."* Client interview 8

In some pharmacies there was a separate queue for CCMDD clients, which they felt could expose their HIV-positive status to others, despite other chronic medication also being collected through CCMDD.

> *"Where I collect, there is our [CCMDD] queue and then there is a queue for everyone else. That is why I say sometimes they would say "these people are coming to collect their thing [ART]".* Focus group 1, Participant 1

Furthermore, integrating HIV treatment with everyday life felt threatening to some clients. One declined referral to CCMDD because he was worried about revealing his HIV status and experiencing stigma.

> "Interviewer: *Okay, why don't you like going there [private pharmacy in CCMDD]?*
>
> Participant: *"Uhm… because this thing [collecting ART] is confidential. Imagine, since I'm working at the mall, how many people who live by my house come there? … Imagine a lady who stays by my place and sees me when I go to this. This is a secret... So, I would prefer to collect them here [at the clinic], because I don't want to feel embarrassed*." Client interview 3

For peer review only - http://bmjopen.bmj.com/site/about/guidelines.xhtml

In general, clients understood that CCMDD was for stable patients, and associated being referred into CCMDD as *meaning* that they had been taking ART well. Some used this to motivate their own adherence.

> *"No one wants to go back to the clinic anymore, and I think that is why we are taking our medication so well... When the nurse showed me my results, I was relieved that I will be going back [to CCMDD]."* Focus group 2, Participant 9

Most clients also understood that quicker ART collection in CCMDD came at the cost of receiving less clinical oversight at the pick-up point, but understood that they could return to the clinic if they were unwell or needed additional care.

> *"It's quicker to just collect, because you know how you are taking your treatment… because when all these things [measuring vital signs, seeing a nurse] are done, you will be delayed."* Focus group 2, Participant 8

> *"…but at [the private pharmacy] you take your medication and leave, and it's good. When you have a problem, you come to the clinic."* Focus group 2, Participant 9

However, two clients liked having their vital signs monitored and the opportunity to talk to nurses in clinic.

> *"They once said we can now get them [ART] outside... I don't want that, I am fine here [at the clinic]… You weigh, check blood pressure and then go to the nurse to collect medication and she will look at your results and tell you everything is going well, or you are lacking here and you are lacking here."* Client interview 16

While successfully collecting ART in CCMDD required new knowledge and skills (*competencies*), some clients' previous experience of buying from pharmacies was helpful.

> *"Ok, so [the first time I collected from CCMDD] I got in there and looked around to see where I'm supposed to go, because I'm used to just buying and then leaving. Ok, I saw that I need to go [to] the dispensary because I am coming to collect and I didn't have a problem I did everything well and I found a good person who was full of jokes."* Client interview 18

Despite the general benefits of CCMDD, a few staff and clients also reported occasional *material problems* with the implementation of the programme (Table 3). These included delays

For peer review only - http://bmjopen.bmj.com/site/about/guidelines.xhtml

in receiving reminder SMS, the client's ART not being found at the pick-up point, inflexible pick-up dates at some pharmacies, with pickup points sending ART back to the central depot, if clients were a few days late, and some pharmacies placing restrictions on when ART could be collected, despite being open for other services. This inflexibility meant that some employed clients still had difficulties collecting their ART in CCMDD (Table 3). At the time of the interviews, several pick-up points had reached capacity and were no longer accepting referrals, meaning that some clients had no pick-up points near to their homes. Staff also reported some barriers to registering people into CCMDD. Referral criteria require a valid identification card or passport, which excluded some clients. Furthermore, isoniazid prophylaxis therapy, a daily tablet to prevent tuberculosis, is not available in CCMDD, meaning clients could not be referred until they had completed the recommended twelve month prophylactic course. Lastly, the electronic prescription system for CCMDD was occasionally offline, which could make CCMDD registration more time consuming (Table 3).

Table 3. Challenges with implementation of CCMDD

Issue	Quotes
**Barriers to registering patients in CCMDD**	
Need for ID card	*"If a patient wants to be registered on CCMDD, strictly that patient must have a South African ID [or foreign passport] and every time the patient is going to collect the medication, they have to positively identify themselves using their ID. Something that we are not doing in the clinics. Some patients are not happy with that, ID's do get lost from time to time and then once the ID is not there, then they cannot give you that package at the pick-up point, then the patient would be forced to come back to the clinic… [and] then they get de-registered in the process."* Staff interview 4*
Problems with electronic prescription system	*"The online registration system crashes quite often so there's days where the nurses can't get online, they just have to register manually. It creates a problem because online registration is a lot quicker, so on the days when they have to do manual then they are backed up for quite a while, and then it just makes the work a lot more."* Staff interview 6
TB prophylaxis	*"There's a category of patients that can be stable on ARV's but still don't qualify for CCMDD. A typical point in example, if at any given point you decide to put this patient on IPT, which is Isoniazid Preventive Therapy for TB, they don't take that patient. You see, for the patient to qualify for CCMDD they must be stable, but now CCMDD will not take a patient that is on IPT. So we have to keep that patient up until they finish the course of IPT before you can enroll them on CCMDD."* Staff interview 4<br><br>*"they said because I am still taking the ones for protecting me against TB and I will finish them in August… after that I will hear from them*

For peer review only - http://bmjopen.bmj.com/site/about/guidelines.xhtml

	[about referral to CCMDD]" Client interview P 21.
**Problems with CCMDD implementation**	
SMS	*"I will receive the text message the following day in the afternoon, after I collected today. I never receive the text message [on time]. I receive it after."* Focus group 1 Participant 3†
ART not being found at the pick-up point	*"I once received an SMS and I went there [to the pick-up point] the very same day I received the SMS, and I got there and they said my name is not there [on the list of people with ART to collect]. I then came here [to the clinic], and I got my treatment here. Fortunately, I had time on that day to end up coming here."* Focus group 2 Participant 4
Inflexible pick-up dates	*The service providers are very strict when it comes to appointment dates, very strict. You get given a date and then you are expected to collect your parcel within 48 hours. After those two days then the medication will be sent back… and they get de-registered as a result. But here in the clinic they can miss the date by a day or 2 or 3 or 4 or 5, it's not that much of an issue.* Staff interview 4<br><br>*"It was my fault because I came after two days and I found that my treatment has been sent back [to the depot]."* Focus group 1 Participant 6
Restricted ART pick-up times	*"The problem with [this community pharmacy] is that they don't issue treatment on weekends. So, since we are working, we don't have time because if I didn't come to work they will deduct a certain amount from my salary. When you go there in the afternoon, they tell you that they don't issue medication at the certain times. Which means I will not be getting my treatment whereas it is very important to me."* Focus group 1 Participant 9
Pick-up points reaching capacity	*"Most of the pick-up points were full, you know. They were full and some of the participants collect their medication far from where they stay, far from where they work you know. Jah, I think those were the disadvantages."* Staff interview 2

DISCUSSION

In this study, clients and healthcare workers identified several problems with collecting ART from HIV clinics in Durban, South Africa. Using 'theories of practice', we showed how these problems interact with, and impact on, engagement in HIV care. We present one of the first qualitative assessment of the largest differentiated ART delivery programme in the world, CCMDD, and show how it overcame some of these problems, helped to normalise HIV treatment and reduced the impact of collecting ART on other social practices in the clients lives. However, several challenges and inconsistencies remain in the implementation of CCMDD.

Clinic based services

We found several barriers to engagement in clinic-based services, which are similar to findings

For peer review only

from other studies. In a qualitative systematic review of 59 studies regarding long-term ART adherence and engagement in care in Africa, poor clinic services were identified as a major theme, with long waiting times, inflexible services and rigid and demanding policies all leading clients to disengage from care.[11] We found that these material challenges interacted with other social practices, such as employment, and also other components of the social practice of engagement in HIV services. For example, in our study and in another study from South Africa, rushed appointments impeded communication with nurses, meaning there was no time for healthcare workers to equip patients with the competencies required to engage with HIV services.[12] Furthermore, we found that healthcare workers were overburdened, sometimes disrespectful, and did not always maintain confidentiality, as has been seen in many other studies.[11] Clinics that separated HIV services from other care also threatened clients confidentiality, therefore exposing clients to stigma, which has been widely reported as impacting negatively on engagement in HIV care.[13,14]

CCMDD

We found that CCMDD changed the ‘materiality’ of ART provision, leading to clear practical benefits for clients. Similar findings have been reported in qualitative assessments of community adherence clubs in Malawi, Mozambique, Kenya and South Africa, where reductions in clinic visits, waiting times, and transport costs,[15–19] reduced disruption to other social practices. In Malawi, Kenya and Zimbabwe, clients had more time to attend their businesses and farming,[17,19] and avoided taking days off work.[16,19] We also found that a major benefit of CCMDD was reduced disruption to employment, although some working clients encountered problems with restricted ART collection times in a few private pharmacies.

CCMDD also impacted on the meanings of engagement in HIV care. Attending private pharmacies was less burdensome, less obviously associated with being HIV positive, and more easily integrated with everyday life, thereby helping to normalise ART collection. In Kenya, integrated adherence clubs for non-communicable diseases and HIV were reported to reduce stigma, by normalising HIV to be like other chronic conditions.[16] However, we found multiple reports of preferential treatment by private pharmacy staff towards paying customers, suggesting some ongoing discrimination towards public sector CCMDD clients. Some clients also remained concerned that their HIV status could be revealed when collecting at community pick-up points, where they could be more likely to see neighbours or people they knew. In studies of community adherence clubs, clients were mainly concerned about revealing their HIV status to other group members, and therefore created strict rules around maintaining group

For peer review only - http://bmjopen.bmj.com/site/about/guidelines.xhtml

confidentiality.[15–18] The risk of other adherence club members breaching confidentiality was offset by increased peer support from within the group.[12,15,17,19] In contrast, clients in CCMDD did not report benefits of peer support, but also did not have to negotiate group dynamics when collecting ART, as they tended to collect individually at pick-up points. Lastly, referral into CCMDD provided a new positive meaning to ART collection, and was seen as a reward and incentive to take ART well. Similarly, adherence clubs clients felt like staff were trusting them to manage their own HIV, and saw being sent back to the clinic as a punishment or failure to adhere to treatment.[12]

Attending CCMDD pickup points also required clients to develop new skills and competencies. When the system worked well, clients generally found it easy to navigate, especially as pick-up points were often in familiar locations. However, as with other differentiated ART delivery programmes,[15–18] there were several reports of implementation problems that clients had to negotiate, such as delayed SMS reminders, ART not being available at the pickup point, and a few pharmacies placing restrictions on ART pick-up times. Furthermore, some of the eligibility criteria for CCMDD seemed to contradict other stated aims of the healthcare system. For example, the need for an identification card could exclude the most vulnerable clients, while not providing tuberculosis prophylaxis within CCMDD could hamper tuberculosis control measures.[20]

Strengths and limitations

This is one of the first qualitative studies to assess the CCMDD programme, the largest differentiated ART delivery model in the world. Our analysis is strengthened by the use of ‘theories of practice’ which highlighted the relationship between the materialities of ART provision, and how changes in these impacted on engagement in HIV care as well as clients’ broader lives. Our interviewers were not involved in CCMDD or clinical patient care, which reduces the potential for social desirability bias. Limitations of our study include the use of a single urban clinic, and the use of clinical trial participants, meaning our findings may not be generalisable to other settings, such as rural areas. While our purposive sampling aimed to include a range of clients, clients who felt more positive towards CCMDD may have been more likely to participate. Lastly, the CCMDD programme is adapting quickly, and some of the early implementation problems that we report (e.g. pick-up points reaching capacity) may now have been resolved.

For peer review only - http://bmjopen.bmj.com/site/about/guidelines.xhtml

Implications

Our findings support the ongoing rollout of CCMDD, while highlighting areas where improvements are needed. Better regulation of private pharmacies could ensure consistent implementation of less restrictive ART collection times, and allow development of strategies to better protect confidentiality and prevent CCMDD clients from receiving inferior care. Increasing the flexibility of the ART collection date window could also increase clients' autonomy to collect on a day that suits them in CCMDD. Steps to reduce stigma at a broader societal level are needed, as fear of disclosure remained a barrier to engagement in both clinic-based services and CCMDD. Further research is needed to establish the impact of CCMDD on clinical outcomes such as viral suppression and retention in care, and whether it is cost-effective. Of note, cost-effectiveness studies should attempt to capture the wider non-healthcare system costs and benefits for employed people, given that CCMDD may be particularly beneficial to them. Lastly, we also identify several areas that could be improved in clinic-based HIV services, including less restrictive opening times, more respectful treatment of clients, and better confidentiality.

Conclusion

In conclusion, we highlight how the material changes to ART provision in CCMDD facilitated engagement with HIV services, reduced disruption in clients' everyday lives, and helped to normalise HIV care. Improvements to the implementation of CCMDD could allow even greater benefits, and contribute towards achieving universal ART through more client-centred healthcare services.

COMPETING INTERESTS

The authors have no competing interests to declare.

ACKNOWLEDGEMENTS

We would like to thank Farzana Osman for descriptive analysis of participants' demographics. Funding: The STREAM study was funded by the US National Institute for Health (AI124719-01). JD is funded by the Wellcome Trust PhD Programme for Primary Care Clinicians (203921/Z/16/Z). LM was funded by DST-NRF Centre of Excellence for HIV Prevention (UID96354). AG is supported by the South African Medical Research Council. STC was supported by funding from the National Institute for Health Research (NIHR) Health Protection

For peer review only - http://bmjopen.bmj.com/site/about/guidelines.xhtml

Research Unit in Healthcare Associated Infections and Antimicrobial Resistance at University of Oxford in partnership with Public Health England (HPRU-2012-10041).

The support of the DST-NRF Centre of Excellence for HIV Prevention towards this research is hereby acknowledged. Opinions expressed, and conclusions arrived at, are those of the authors and are not necessarily to be attributed to the DST-NRF Centre of Excellence for HIV Prevention.

AUTHORS' CONTRIBUTIONS

JD, AG, HN, PKD and NG conceived and designed the study. LM and HPS conducted interviews and focus group discussions, and transcribed and translated the data. JD, NG, HN and AG supervised data collection. JD and LM coded and analysed the data. AG, STC, GH and CB supervised data analysis. JD drafted the manuscript. JD, LM, AG, HS, STC, GH, CB, HN, PKD and NG critically reviewed and edited the manuscript and consented to publication.

DATA AVAILABILITY STATEMENT

No data are available

REFERENCES

1 Joint United Nations Programme on HIV/AIDS (UNAIDS). Communities at the Centre: Global AIDS Update 2019. Geneva, Switzerland: 2019. https://www.unaids.org/en/20190716_GR2019_communities

2 Grimsrud A, Barnabas R V., Ehrenkranz P, *et al.* Evidence for scale up: the differentiated care research agenda. *J Int AIDS Soc* 2017;**20**:22024. doi:10.7448/IAS.20.5.22024

3 Grimsrud A, Bygrave H, Doherty M, *et al.* Reimagining HIV service delivery: the role of differentiated care from prevention to suppression. *J Int AIDS Soc* 2016;**19**:21484. doi:10.7448/IAS.19.1.21484

4 Ehrenkranz P, Grimsrud A, Rabkin M. Differentiated service delivery. *Curr Opin HIV AIDS* 2018;**14**:1. doi:10.1097/COH.0000000000000509

5 South African National Department of Health. Adherence Guidelines for HIV, TB and NCDs. Pretoria: 2016.

6 Roberts P. Centralized Chronic Medicine Dispensing and Distribution: A Public/Private Partnership to Increase Access to HIV/Chronic Medication. 2018. http://files.icap.columbia.edu/files/uploads/ICAP_Grand_Rounds_Project_Last_Mile_Slid

For peer review only - http://bmjopen.bmj.com/site/about/guidelines.xhtml

es.pdf

7 Skovdal M, Wringe A, Seeley J, *et al.* Using theories of practice to understand HIV-positive persons varied engagement with HIV services: a qualitative study in six Sub-Saharan African countries. *Sex Transm Infect* 2017;**93**:e052977. doi:10.1136/sextrans-2016-052977

8 Dorward J, Garrett N, Quame-Amaglo J, *et al.* Protocol for a randomised controlled implementation trial of point-of-care viral load testing and task shifting: the Simplifying HIV TREAtment and Monitoring (STREAM) study. *BMJ Open* 2017;**7**:e017507. doi:10.1136/bmjopen-2017-017507

9 Msimango L, Gibbs A, Shozi H, *et al.* Acceptability of point-of-care viral load testing to facilitate differentiated HIV care: a qualitative study of clients and healthcare workers. In: *International AIDS Society*. Mexico City, Mexico: 2019.

10 Attride-Stirling J. Thematic networks: an analytic tool for qualitative research. *Qual Res* 2001;**1**:385–405. doi:https://doi.org/10.1177/146879410100100307

11 Eshun-Wilson I, Rohwer A, Hendricks L, *et al.* Being HIV positive and staying on antiretroviral therapy in Africa: A qualitative systematic review and theoretical model. *PLoS One* 2019;**14**:e0210408. doi:10.1371/journal.pone.0210408

12 Venables E, Towriss C, Rini Z, *et al.* Patient experiences of ART adherence clubs in Khayelitsha and Gugulethu, Cape Town, South Africa: A qualitative study. *PLoS One* 2019;**14**:e0218340. doi:10.1371/journal.pone.0218340

13 Turan B, Hatcher AM, Weiser SD, *et al.* Framing Mechanisms Linking HIV-Related Stigma, Adherence to Treatment, and Health Outcomes. *Am J Public Health* 2017;**107**:863–9. doi:10.2105/AJPH.2017.303744

14 Horter S, Bernays S, Thabede Z, *et al.* "I don't want them to know": how stigma creates dilemmas for engagement with Treat-all HIV care for people living with HIV in Eswatini. *African J AIDS Res* 2019;**18**:27–37. doi:10.2989/16085906.2018.1552163

15 Rasschaert F, Telfer B, Lessitala F, *et al.* A qualitative assessment of a community antiretroviral therapy group model in Tete, Mozambique. *PLoS One* 2014;**9**:1–11. doi:10.1371/journal.pone.0091544

16 Venables E, Edwards JK, Baert S, *et al.* 'They just come, pick and go.' the acceptability of integrated medication adherence clubs for HIV and Non Communicable Disease (NCD) Patients in Kibera, Kenya. *PLoS One* 2016;**11**:1–12. doi:10.1371/journal.pone.0164634

17 Pellecchia U, Baert S, Nundwe S, *et al.* "We are part of a family". Benefits and limitations of community ART groups (CAGs) in Thyolo, Malawi: a qualitative study. *J Int AIDS Soc*

For peer review only

2017;**20**:21374. doi:10.7448/IAS.20.1.21374

18 Prust ML, Banda CK, Nyirenda R, *et al.* Multi-month prescriptions, fast-track refills, and community ART groups: Results from a process evaluation in Malawi on using differentiated models of care to achieve national HIV treatment goals. *J Int AIDS Soc* 2017;**20**:41–50. doi:10.7448/IAS.20.5.21650

19 Bochner AF, Meacham E, Mhungu N, *et al.* The rollout of Community ART Refill Groups in Zimbabwe: a qualitative evaluation. *J Int AIDS Soc* 2019;**22**. doi:10.1002/jia2.25393

20 Tram KH, Mwangwa F, Chamie G, *et al.* Predictors of isoniazid preventive therapy completion among HIV-infected patients receiving differentiated and non-differentiated HIV care in rural Uganda. *AIDS Care - Psychol Socio-Medical Asp AIDS/HIV* 2019;**0121**. doi:10.1080/09540121.2019.1619661

FIGURE 1: SUMMARY OF THEORIES OF PRACTICE FRAMEWORK, REPRODUCED WITH PERMISSION FROM SKOVDAL ET AL., 2017[7]

For peer review only

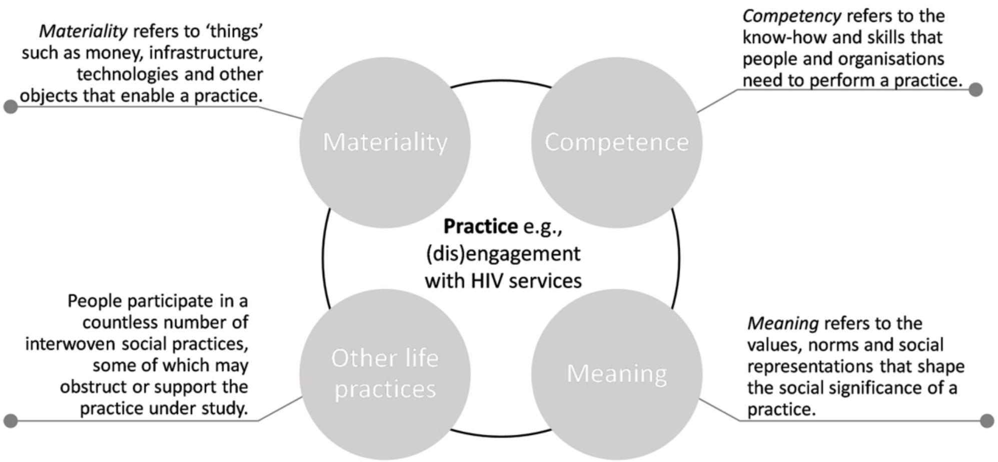

Summary of Theories of Practice Framework, reproduced with permission from Skovdal et al., 2017[7]

363x167mm (300 x 300 DPI)

Table S1: Coding frame based on Framework of Social Practice

Code	Description	Quote
**MATERIALITIES:** Physical 'things' that impact on engagement in HIV services		
**Characteristics of HIV services**	The physical aspects of HIV services that determine access	
Waiting times	Descriptions of waiting times in HIV services	*"When you are coming to the clinic, you must cancel all the plans you have and tell yourself that you will spend the rest of the day there. Because chances are you will finish late in the afternoon."* Focus group 2, Participant 1<br><br>*"The clinic gets very full, you will wait there the whole day."* Focus group 3, Participant 3<br><br>*"I think collecting at [the private pharmacy] is quicker than here, when I came back here at the clinic I waited for a good two hours."* Focus group 1, Participant 7<br><br>*"They [patients] like CCMDD, [because] when they go to the pick-up points there's no queues like here in the clinic. They come in, they go straight to the pharmacies then they get their pack."* Staff interview 4
Numbers of patients	Descriptions of the number of patients receiving treatment	*"They [nurses] are always in a rush and there are a lot of us. Maybe we are more than 100 and there's a queue."* Focus group 1, Participant 4<br><br>Interviewer: *"Ok, so the first time you went to the hall [CCMDD community pick-up point], how was it?"* Participant: *"It was nice because there was not a lot of us, I would just take my ID out and then take my treatment and leave."* Focus group 2, Participant 2.

Code	Description	Quote
Staffing levels	Descriptions of staffing levels in HIV services	*"There is not enough staff [in the clinic], everyone is rushing to finish... Maybe only three nurses are working, and they are seeing all these people. You are also rushing and she is also rushing and you are thinking as a patient that you left a lot of people outside [waiting to be seen after you]. So adding more staff would help."* Client interview 3
Flexibility of HIV services	Descriptions of (in)flexibility of HIV services with regard opening hours and ability to change appointment days	*"Collecting on weekends would be a great idea, because you will not have an excuse especially if you are not working. Even at work they will have a problem if you have a specific day where you don't come to work or leave early [to collect ART]."* Focus group 2, Participant 1<br><br>*"Collecting… [in CCMDD] saves a lot of time, you don't spend 5 minutes. It doesn't get full, you can come anytime if it's still open. You just get in there take your medication and leave."* Focus group 3, Participant 5
Accessing other medical care apart from ART	Descriptions of how the ability to access other, non-ART related care influenced engagement with HIV services	*"It's quicker to just collect [ART] because you know how you are taking your treatment… When all these things [measuring vitals] are done you will be delayed."* Focus group 2, Participant 8<br><br>*"The problem with it [CCMDD] is that you only come to collect your medication and then leave, they don't do other things as well. Like checking your blood pressure and other things to know how your health is."* Client interview 5
Maintaining confidentiality	Descriptions of how the design of the service impacts on confidentiality	*"But at the nearby clinics, most of the nurses just display everything [ART] in front of everyone… there is no privacy in these clinics."* Focus group 1, Participant 8

For peer review only - http://bmjopen.bmj.com/site/about/guidelines.xhtml

Code	Description	Quote
		"When your neighbors see you sitting there they will start to ask you questions. Even when they don't know why you are there, but they will know at the end why." Focus group 1, Participant 6
ART not being available in facility or pickup point	Descriptions of attending services but ART not being available	*"Sometimes you find that there is no treatment [in the clinic] and people would be scheduled for another date when the treatment is available."* Client interview 21
Distance to HIV services	Descriptions of the distance and travel to HIV services affecting engagement in care	*"There are people who live far [from the clinic]… you find that they don't even have money for transport."* Client interview 6
Barriers to engagement with CCMDD	Descriptions of factors that stop people from being referred into CCMDD or continuing to collect there (see Table 3)	See Table 3 for specific quotes
**Poverty**	The dynamics of lacking financial and material resources (e.g. food, other medication to manage side effects) that would support engagement with HIV services.	*"Fetching medication is problematic. You find that you don't have money, you need to fetch your medication and you don't have it [money]."* Client interview 1
**COMPETENCIES:** Knowledge and skills that impact on engagement in HIV services		
**Communication between healthcare workers and patients**	The role of healthcare workers in communicating competencies and also clinical information about HIV and treatment to patients	*"Patient education… should be constant, it should be regular, regular talking with the patients, teaching patients how to live positively, considering things like the use of condoms, proper dieting and… the function of adherence."* Staff interview 4<br><br>Interviewer: *"When you ask the nurse about your results, what do they say?"*

For peer review only - http://bmjopen.bmj.com/site/about/guidelines.xhtml

Code	Description	Quote
		Participant: *"You even get afraid to ask, because they don't have time, they are always rushing to see the next person."* Focus group 3, Participant 4
Healthcare worker attitude	Descriptions of the importance of healthcare worker attitude in imparting these competencies towards patients	*"They [healthcare workers] must not judge us, they must do their job and what they were employed to do. Also, they need to learn to communicate, nurses can't communicate with people. You make one small mistake [and] they shout at you and talk to you the way they like."* Client interview 19
**To manage stigma**	Knowledge and skills needed to manage the (non)disclosure of one's HIV status to others, and to deal with stigma from others including partners, family members and the wider community.	*"I didn't want to go to the clinic by where I live because I was afraid of people's eyes. Because when you start this [ART] people look at you in different way."* Client interview 20
**To negotiate problems with HIV services**	Descriptions of strategies and skills that people have developed to mitigate problems with HIV services	*"You see, I make it a point that when it's my appointment date I wake up very early to get to the clinic… I will be here for the whole day… because there are a lot of people… You must wait that long and sometimes you get so tired and you wish you can just leave. What can we say? We must wait because it's our lives."* Client interview 12<br><br>*"If you don't have a day-off on that day you can send someone to collect the medication for you."* Client interview 1
**Social support**	The use of family and or the wider community as a source of knowledge to help maintain engagement with HIV services	"*[After starting ART] I did have a bad dream on the first day... But after that there were no problems. I asked other people, and they told me that they did have side-effects on the first day, you will have bad dreams and I didn't have a problem with that, it passed. Because when I asked other people they said, yes, it happens. But the ARVs are no longer the same as the previous ones, these ones are*

For peer review only - http://bmjopen.bmj.com/site/about/guidelines.xhtml

Code	Description	Quote
		better because you only take one pill at night. Yes, I continued I took the medication.” Client interview 1.
**Maintaining a healthy lifestyle**	Knowledge and skills needed to maintain recommended lifestyle changes for people living with HIV	*“They were advising us that we shouldn’t tell ourselves that we are going to die you see… that if you are person living with HIV you practice safe sex and not sleep around and try to eat healthy things in life. If you smoke reduce a bit and [reduce] drinking. General things were being spoken that we need to deal with.”* Client interview 10
**MEANINGS:** The meanings that are attached to HIV and engagement with HIV services		
**Stigma**	Prejudice and discrimination experienced by people living with HIV. Can be internal (‘self-stigma’) or external (enacted by others) and both perceived or actual.	*“[if there is a separate queue for ART] it’s easier to be discriminated against, because they know what you are here for, so it’s better for a person not to know what you are queuing for.”* Focus group 1, Participant 4<br><br>*“I wish they could make this disease [HIV] like flu, and not something scary. That is the reason why people don’t even want to come close to you.”* Focus group 1, Participant 7<br><br>*“My concern is the stigma we have as infected people, we get treated as people who don’t live in this world. So, we try to hide ourselves.”* Focus group 2, Participant 4.
**Normalisation of HIV**	The portrayal of HIV as a common and normal experience share with many others, that does not have to affect health if ART is taken.	Interviewer: *“When you found out [that you were HIV-positive] … how did you feel about that?”*<br>Participant: *“I didn’t feel like a different person, I didn’t see any difference, I saw myself as a normal person who needs to take care of herself and take her medication properly so life can go on. I didn’t feel any change and I wasn’t sick at all.”* Client interview 5

For peer review only - http://bmjopen.bmj.com/site/about/guidelines.xhtml

Code	Description	Quote
**Meanings of CCMDD**	Descriptions of the meaning associated with being referred to CCMDD including reasons for being referred	*"They transferred me to CCMDD because I was taking my treatment properly and I also know that I was taking my treatment well."* Focus group 2, Participant 7
**Public and private treatment**	The meanings associated with receiving government funded versus private care	*"Sometimes you wait for other [customers] to buy and they [private pharmacy staff] will serve the other people before you. Even if you are in the queue, it's like they are sifting us [public CCMDD clients] from the crowd."* Focus group 1, Participant 5<br><br>*"They [private pharmacy staff] were paying attention to the customers who were coming to buy. Because we don't come with money, we only come with our [CCMDD] cards and ID's [so] they were not paying attention to us."* Client interview 8
**Experience of HIV and treatment**	Having had personal experience, or having witnessed in others, the effects of HIV and life-saving treatment. Having faith in treatment and HIV services in restoring health and life	*"As for me, even when I walk on the streets I feel healthy and confident because I know I am taking my medication properly."* Focus group 2, Participant 9
**OTHER LIFE PRACTICES:** Other repeated activities of daily life that clients engage in that impact on engagement in HIV services		
**Employment**	The duties and daily activities of employment and formal and informal work	*"I even go after work, I knock off at 4 and go there [CCMDD pick-up point] and take my medication. Even during my break time, I go, or when I am swamped I ask [my supervisor] and quickly rush there take my medication and come back… What I love [about CCMDD] is that I don't need to take a day off at work to collect medication at the clinic and wait for the whole day. Now at work I ask for that 20 minutes and go there [to CCMDD]. It's fine. I don't skip work, and there is no one that sees me."* Client interview 12

For peer review only - http://bmjopen.bmj.com/site/about/guidelines.xhtml

Code	Description	Quote
		"I think it [CCMDD] is good, because clinics get full. It's better because if you work you can quickly go there [the community pick-up point], collect and come back. I think it's a good method." Client interview 4
**Family life**	Activities of daily life that revolve around family	*"Last time [I had to collect ART from the clinic] you know what happened? I had to send my wife. I'd got a part time job, so I couldn't manage to come. [So I asked her], 'Please just go with my card to collect my medication'. She doesn't want to come back here [to the clinic] ever again. Because of the time that she spent [waiting for treatment]."* Client interview 14

For peer review only

For peer review only - http://bmjopen.bmj.com/site/about/guidelines.xhtml

Understanding how community antiretroviral delivery programmes influence engagement in HIV care: a qualitative assessment of the Centralised Chronic Medication Dispensing and Distribution programme in South Africa

Supporting Information

Table of Contents **Page**

1. Participant in-depth interview topic guide 1

2. Staff in-depth interview topic guide 3

3. Participant focus group topic guide – CCMDD 5

For peer review only

For peer review only

Patient and service provider perspectives regarding point of care viral load testing and differentiated care: an ancillary study to the STREAM study

Participant in Depth Interview Topic Guide

The overarching objectives of this interview topic guide are to:

- Understand participants experience of laboratory and point of care viral load testing
- Understand participants experience of receiving care from an enrolled nurse compared to a professional nurse
- Understand participants experience of receiving HIV care at clinics, community based organisations and pharmacies.

The interview should last approximately 30 to 60 minutes. The questions are indicative of the topics covered and will be shaped by participants' discussions and concerns. The interview will be conducted by a skilled Zulu facilitator and recorded without participant's name or personal details, but with their consent and permission for further analysis.

Introduction and background experience of HIV care

Tell me a little about yourself, your age, where you stay, what you do to get by, and where you have been getting your treatment from.

Laboratory and point of care testing

Tell me about the last time you had a blood test as part of your HIV care. When did you get the results?

How did you feel while you were waiting for the results?

Tell me about the results that you were given. How did you feel about these results?

If you had point of care blood taken, what were the good things and what were the bad things about this approach?

What would you want us to do differently with blood testing? Why do you think this would help?

Antiretroviral therapy collection at community centres and pharmacies

Tell me about the last time that you collected your antiretrovirals (ARVs). Where did you go, who did you talk to and how was the experience?

What were the good things about collecting treatment there, and what were the bad things? If you ever collected treatment at a community centre or community pharmacy, can you tell me the story of the first time you went there?

What other things are going on in your life which make it easier or harder to collect your treatment?

Enrolled nurse and professional nurse care

Can you tell me about a time when you felt unwell and told your problem to a nurse? What happened? How did you feel about the care you received?

Tell me about the last time you came to clinic and saw a nurse. What did the nurse do for you that day?

For peer review only - http://bmjopen.bmj.com/site/about/guidelines.xhtml

If you saw an enrolled nurse, rather than a professional nurse, how did you feel about the care you received from the enrolled nurse?

What were the good and bad things about seeing an enrolled nurse?

Wrap Up

Have you got any other ideas that may help improve HIV care services?

For peer review only

For peer review only - http://bmjopen.bmj.com/site/about/guidelines.xhtml

Patient and service provider perspectives regarding point of care viral load testing and differentiated care: an ancillary study to the STREAM study

Staff in Depth Interview Topic Guide

The overarching objectives of this interview are to:

- Understand staff experience and views on laboratory and point of care viral load testing
- Understand staff experience and views regarding care provision by an enrolled nurse compared to a professional nurse
- Understand staff experience and views regarding care provision at clinics, community based organisations and pharmacies.

The interview should last approximately 30 to 60 minutes. The questions are indicative of the topics covered and will be shaped by the discussions and interviewees concerns. The interview will be conducted by a skilled facilitator and recorded without participant's name or personal details, but with their consent and permission for further analysis.

Introduction and background experience of HIV care

Tell me a little about yourself, where you have worked in the past, where you work now and what your main role at work is.

Laboratory and point of care testing

Tell me about any point of care (POC) tests that are used in your clinic/area of work, and your experience of using them.

What do you like and not like about each of these POC tests?

How could these POC tests be improved?

In your experience, what are the most important features of POC tests?

Tell me about the last time you told a patient that they had a high viral load. If this was a POC test, do you think giving the result on the day made any difference? What difference would there have been if it was a laboratory test?

If this was a laboratory test, what difference would there have been if it was a POC test?

Tell me the ways that you think POC testing could improve HIV care?

ART collection at community centres and pharmacies (CCMDD)

Tell me about your experience of the Centralised Chronic Medicines Dispensing and Distribution (CCMDD) system. What are the good things and bad things about it?

Tell me the main reasons that patients like CCMDD.

Tell me the main reasons that patients don't like CCMDD.

How do you think the CCMDD system could be improved?

For peer review only - http://bmjopen.bmj.com/site/about/guidelines.xhtml

Enrolled nurse and professional nurse care

What do you feel are the most important roles that enrolled nurses currently perform in the HIV programme?

What do you feel are the most important roles that professional nurses currently perform in the HIV programme?

Do you think any professional nurse roles could be done by enrolled nurses? Which ones and why/why not?

Are there any roles that professional nurses DON'T currently perform that you think they could or should do?

In our study, enrolled nurses have been seeing stable patients. What advantages and disadvantages do you think this approach has?

Are there any ways that you think we could improve this approach?

Wrap Up

Have you got any other ideas of how POC testing, community ART delivery/CCMDD or using enrolled nurses in innovative ways may help improve HIV care services?

For peer review only

For peer review only - http://bmjopen.bmj.com/site/about/guidelines.xhtml

For peer review only

Patient and service provider perspectives regarding point of care viral load testing and differentiated care: an ancillary study to the STREAM study

Participant Focus Group Topic Guide - CCMDD

We will arrange to meet participants enrolled in the study in a private location at or near the clinic to participate in a focus group. Between 5-10 participants will be in each focus-group.

The overarching objectives of this focus group topic guide are to:

- Understand participants experience of receiving HIV care at clinics, community based organisations and pharmacies.

The focus groups should last approximately 45-75 minutes. The questions are indicative of the topics covered and will be shaped by participants' discussions and concerns. The group will be facilitated by a skilled Zulu facilitator and recorded without participants' names or personal details, but with their consent and permission for further analysis

Introduction and background experience of HIV care

Tell us a little about your lives, your age, where you stay, what you do to get by, and where you have been getting your treatment from.

ART collection at community centres and pharmacies

Can someone who had a problem getting their treatment in the last few months tell us the story about what happened?

Did anyone else in the group experience something similar and manage to find a way around it – can you describe this please?

Is there anything that the CAPRISA or CDC clinic could have done to help you overcome these problems?

Does anyone have examples of other strategies that you or other people use to fit collecting medication into your everyday life?

Some people have been able to collect their treatment from community pharmacies and community centres. Can someone who did this tell us about the first time they went to collect their treatment there, where did you go, who did you talk to and how was the experience?

Does anyone else have a similar story? What were the good things about collecting treatment there, and what were the bad things? How did you feel when staff first told you that you could collect treatment at the community centre or pharmacy? Can anyone tell us about what happens when they come back to the clinic for review every 6 months?

Wrap Up

Have any of you got any other ideas that may help improve HIV care services?

For peer review only - http://bmjopen.bmj.com/site/about/guidelines.xhtml

BMJ Open

Understanding how community antiretroviral delivery influences engagement in HIV care: a qualitative assessment of the Centralised Chronic Medication Dispensing and Distribution programme in South Africa

Journal:	*BMJ Open*
Manuscript ID	bmjopen-2019-035412.R1
Article Type:	Original research
Date Submitted by the Author:	12-Feb-2020
Complete List of Authors:	Dorward, Jienchi; University of Oxford, Nuffield Department of Primary Care Health Sciences; Centre for the Aids Programme of Research in South Africa,<br>Msimango, Lindani; Centre for the Aids Programme of Research in South Africa,<br>Gibbs, Andrew; South African Medical Research Council, Gender and Health Research Unit; University of KwaZulu-Natal, Centre for Rural Health<br>Shozi, Hlengiwe; Centre for the Aids Programme of Research in South Africa,<br>Tonkin-Crine, Sarah ; University of Oxford, Nuffield Department of Primary Care Health Sciences; University of Oxford, 5 NIHR Health Protection Research Unit in Healthcare Associated Infections and Antimicrobial Resistance<br>Hayward, Gail; University of Oxford, Nuffield Department of Primary Care Health Sciences<br>Butler, Christopher C.; University of Oxford, Nuffield Department of Primary Care Health Sciences<br>Ngobese, Hope; Ethekwini Municipality, Health Unit<br>Drain, Paul; University of Washington, Departments of Global Health, Medicine and Epidemiology<br>Garrett, Nigel; Centre for the Aids Programme of Research in South Africa, ; University of KwaZulu-Natal, Discipline of Public Health Medicine, School of Nursing and Public Health,
**Primary Subject Heading**:	HIV/AIDS
Secondary Subject Heading:	Health services research, General practice / Family practice, Public health, Qualitative research, Global health
Keywords:	Public health < INFECTIOUS DISEASES, International health services < HEALTH SERVICES ADMINISTRATION & MANAGEMENT, HIV & AIDS < INFECTIOUS DISEASES, PRIMARY CARE

For peer review only - http://bmjopen.bmj.com/site/about/guidelines.xhtml

I, the Submitting Author has the right to grant and does grant on behalf of all authors of the Work (as defined in the below author licence), an exclusive licence and/or a non-exclusive licence for contributions from authors who are: i) UK Crown employees; ii) where BMJ has agreed a CC-BY licence shall apply, and/or iii) in accordance with the terms applicable for US Federal Government officers or employees acting as part of their official duties; on a worldwide, perpetual, irrevocable, royalty-free basis to BMJ Publishing Group Ltd ("BMJ") its licensees and where the relevant Journal is co-owned by BMJ to the co-owners of the Journal, to publish the Work in this journal and any other BMJ products and to exploit all rights, as set out in our licence.

The Submitting Author accepts and understands that any supply made under these terms is made by BMJ to the Submitting Author unless you are acting as an employee on behalf of your employer or a postgraduate student of an affiliated institution which is paying any applicable article publishing charge ("APC") for Open Access articles. Where the Submitting Author wishes to make the Work available on an Open Access basis (and intends to pay the relevant APC), the terms of reuse of such Open Access shall be governed by a Creative Commons licence – details of these licences and which Creative Commons licence will apply to this Work are set out in our licence referred to above.

Other than as permitted in any relevant BMJ Author's Self Archiving Policies, I confirm this Work has not been accepted for publication elsewhere, is not being considered for publication elsewhere and does not duplicate material already published. I confirm all authors consent to publication of this Work and authorise the granting of this licence.

For peer review only

TITLE: UNDERSTANDING HOW COMMUNITY ANTIRETROVIRAL DELIVERY INFLUENCES ENGAGEMENT IN HIV CARE: A QUALITATIVE ASSESSMENT OF THE CENTRALISED CHRONIC MEDICATION DISPENSING AND DISTRIBUTION PROGRAMME IN SOUTH AFRICA

AUTHORS

Jienchi Dorward[1,2§], Lindani Msimango[1], Andrew Gibbs[3,4], Hlengiwe Shozi[1], Sarah Tonkin-Crine[2,5], Gail Hayward[2], Christopher C Butler[2], Hope Ngobese[6], Paul K Drain[7,8,9], Nigel Garrett[1,10]

AFFILIATIONS

1 Centre for the AIDS Programme of Research in South Africa (CAPRISA), University of KwaZulu–Natal, Durban, South Africa.
2 Nuffield Department of Primary Care Health Sciences, University of Oxford, Oxford, UK.
3 Gender and Health Research Unit, South African Medical Research Council, Pretoria, South Africa.
4 Centre for Rural Health, School of Nursing and Public Health, University of KwaZulu-Natal, Durban, South Africa.
5 NIHR Health Protection Research Unit in Healthcare Associated Infections and Antimicrobial Resistance, University of Oxford, Oxford, UK
6 Ethekwini Municipal Health Department, Durban, South Africa
7 Department of Global Health, Schools of Medicine and Public Health, University of Washington, Seattle, USA.
8 Department of Medicine, School of Medicine, University of Washington, Seattle, USA.
9 Department of Epidemiology, School of Public Health, University of Washington, Seattle, USA.
10 Discipline of Public Health Medicine, School of Nursing and Public Health, University of KwaZulu-Natal, Durban, South Africa.

[§] Corresponding author: Dr Jienchi Dorward

Nuffield Department of Primary Care Health Sciences, University of Oxford,
Gibson Building 1st Floor, Radcliffe Observatory Quarter, Woodstock Road, Oxford, OX2 6GG
Email: Jienchi.Dorward@phc.ox.ac.uk

For peer review only

E-mail addresses of authors:

JD: jienchi.dorward@phc.ox.ac.uk

LM: lindani.msimango@caprisa.org

AG: Andrew.Gibbs@mrc.ac.za

HS: Hlengiwe.Shozi@caprisa.org

STC: sarah.tonkin-crine@phc.ox.ac.uk

GH: gail.hayward@phc.ox.ac.uk

CB: christopher.butler@phc.ox.ac.uk

HN: hope.ngobese@durban.gov.za

PD: pkdrain@uw.edu

NG: nigel.garrett@caprisa.org

Keywords: differentiated care; community; antiretroviral therapy; HIV; South Africa

STREAM study clinical trial registration: NCT03066128, registered February 2017

Word counts: Abstract 300/300. Main text: 4940/5000

For peer review only

ABSTRACT (300/300)

Introduction

Providing antiretroviral therapy (ART) for millions of people living with HIV requires efficient, client-centred models of differentiated ART delivery. In South Africa, the Centralised Chronic Medication Dispensing and Distribution (CCMDD) programme allows over 1 million people to collect ART from community pick-up points. We aimed to explore how CCMDD influences engagement in HIV care.

Methods

We performed in-depth interviews and focus group discussions with clients receiving ART and health-care workers in Durban, South Africa. We analysed transcripts using deductive thematic analysis, with a framework informed by 'theories of practice', which highlights the *materialities*, *competencies*, *meanings,* and *other life practices* that underpin clients' engagement in HIV care.

Results

Between March-August 2018 we undertook 25 interviews and four focus groups with a total of 55 clients, and interviewed eight healthcare workers. The material challenges of standard clinic-based ART provision included long waiting times, poor confidentiality, and restricted opening hours, which discouraged clients from engagement. In contrast, CCMDD allowed quicker and more convenient ART collection in the community. This required the development of new competencies around accessing care, and helped change the meanings associated with HIV, by normalising treatment collection. CCMDD was seen as a reward by clients for taking ART well, and helped reduce disruption to other life practices such as employment. At private pharmacies, some clients reported receiving inferior care compared to paying customers, and some worried about inadvertently revealing their HIV status. Clients and healthcare workers had to negotiate problems with CCMDD implementation, including some pharmacies reaching capacity or only allowing ART collection at restricted times.

Conclusions

In South Africa, CCMDD overcame material barriers to attending clinics, changed the meanings associated with collecting ART and was less disruptive to other social practices in clients' lives. Expansion of community-based ART delivery programmes may help to facilitate engagement in HIV care.

For peer review only

ARTICLE SUMMARY

Strengths and limitations of this study

- This is one of the first qualitative assessments of the Centralised Chronic Medication Dispensing and Distribution programme (CCMDD), which provides ART to over 1 million people in South African communities.
- Our analysis is strengthened by the use of 'theories of practice', which highlights the different components of the social practice of engagement in HIV care, and how these interact and may be influenced by CCMDD.
- Participants in our study were part of a clinical trial at a large urban clinic, meaning our findings may not be generalisable to other settings, such as rural areas.
- Lastly, the CCMDD programme is adapting quickly, and some of the early implementation problems that we report may now have been resolved.

BACKGROUND

Expanding access to life-saving antiretroviral therapy (ART) for people with HIV has been one of the biggest global health successes of the 21st century. Since 2004, the number of people on ART globally has increased more than ten-fold to 23.3 million, while HIV related mortality has dropped from 1.7 million annual deaths to 0.8 million.[1] ART is now recommended for all people living with HIV, meaning that a further 14.6 million people still require treatment, mainly in low- and middle-income countries.[1] Therefore, ART programmes in these settings will need to continue expanding, while also providing treatment through efficient, convenient services that encourage life-long engagement in HIV care.

Several new models of 'differentiated ART delivery' have been developed, including providing ART through community adherence clubs (where clients meet in a community group to collect ART), delivering ART to decentralized pick up points, and increasing the intervals between ART collection dates using multi-month prescribing.[2] These services aim to provide streamlined, efficient treatment in a client-centred manner for people who are doing well on ART, thereby allowing more resources to be directed towards initiating people on treatment and to managing those who are less well.[3,4]

South Africa currently runs the largest differentiated ART delivery programme in the world as part of the Centralized Chronic Medication Dispensing and Distribution Programme (CCMDD).[5] The CCMDD programme allows people with and without HIV to collect pre-packed

For peer review only - http://bmjopen.bmj.com/site/about/guidelines.xhtml

chronic medications (e.g. ART, antihypertensives, diabetes treatment, lipid-lowering medication,) in the community, instead of having treatment dispensed at clinics. People living with HIV can be referred into CCMDD by their ART clinic if they are non-pregnant, stable on ART and have had two consecutive suppressed HIV viral loads, at least six months apart. In CCMDD, clients collect their treatment every 2 months at local pickup points, including private pharmacies and community-based organizations.[6] Every six months, they return to the clinic for review and re-referral into CCMDD. An annual viral load is also performed at the clinic assessing whether they remain virally suppressed. If clients in CCMDD are unwell, they can return to their clinic at any time for medical attention. CCMDD is a public-private partnership; patients are referred by healthcare workers in government sector clinics, while a private company is responsible for centrally dispensing medication and delivering it to community pick-up points.[5,7] CCMDD was started in 2014 and provides treatment to over 1.7 million people[8]. However, despite its size, there is little published data evaluating how this programme influences engagement in HIV care.

Several different frameworks and theories have been proposed to evaluate factors influencing engagement in HIV care. Skovdal et al [9] propose a theoretical framework around engagement in HIV care as a social practice (Figure 1). 'Theories of practice' conceptualise patterns of behaviour as social practices which depend on *materialities* (e.g. clinic accessibility), *competencies* (e.g. knowing how to access services and/or maintain healthy lifestyle), *meanings* (e.g. HIV-associated stigma, the normalisation of HIV as a common experience), and *other life practices* (e.g. employment).[9] These different components dynamically interact to influence the 'practice' of engagement in HIV care. Here, we aim to use this framework to explore engagement in clinic-based HIV services, and to analyse how CCMDD may influence the social practice of engagement in HIV care.

METHODS

Study design and setting

We undertook semi-structured in-depth interviews and focus group discussions with clients, and interviews with healthcare workers, who were part of the Simplified TREAtment and Monitoring (STREAM) study, a clinical trial of point-of-care HIV viral load testing and task shifting to enrolled nurses (NCT03066128).[10,11] We previously reported qualitative findings from this study regarding the acceptability of point-of-care viral load testing and enrolled nurse care[12]. Here, we focus on findings related to CCMDD.

For peer review only - http://bmjopen.bmj.com/site/about/guidelines.xhtml

Eligible participants in STREAM were adults aged ≥18 years who were stable on first-line ART for 6 months after initiation. At enrolment, participants were randomized 1:1 to receive point-of-care viral load testing and potential task shifting to enrolled nurses, versus standard laboratory viral load testing and professional nurse care. After six months in the study (one year on ART) participants in both arms who had a suppressed viral load and met other eligibility criteria (clinically stable, non-pregnant, CD4 count >200 cells/mm^3)[6] were offered referral into CCMDD.

Once accepted into CCMDD, participants were able to collect treatment every two months at private pharmacies and community-based organisations. The CCMDD system would automatically send SMS-texts to clients' mobile phones to remind them of their ART collection dates. After six months they were reviewed at the clinic by a nurse. The STREAM study took place at the Prince Cyril Zulu Clinic and the adjacent CAPRISA Ethekwini Clinical Research Site, which are situated next to the main transport hub in central Durban, South Africa. Prince Cyril Zulu Clinic serves a large, urban population and was one of the first clinics to implement CCMDD in Durban, as part of a pilot implementation programme.

Participants

We used a purposive sampling frame to select a range of patients including those who received care in CCMDD, who had suppressed and unsuppressed viral loads at any point in the study, and who received point-of-care testing versus standard laboratory testing in the STREAM trial (Table 1). We approached potential participants in person or by telephone after they had exited the STREAM study, explained reasons for performing the research and obtained written informed consent for their participation.

Table 1: Client in-depth interview sampling frame

		**Point-of-care arm**	**Standard-of-care arm**	**Total target**
		Target	Target	
**Viral load result**	**Care provider**			
**< 1000 copies/ml**	CCMDD and/or enrolled nurse	5	5	10
	Professional nurse	5	5	10
**> 1000 copies/ml**	Professional nurse	5	5	10
Total target		15	15	30

CCMDD, Centralised Chronic Medication Dispensing and Distribution programme

For peer review only - http://bmjopen.bmj.com/site/about/guidelines.xhtml

Data collection

Trained research assistants (LM [male], HS [female]) performed interviews and focus groups in IsiZulu or English in a private room in the clinic, using semi-structured topic guides (supplementary material). LM was not previously known to participants. HS was known to patients as she was involved in recruitment and scheduling in STREAM. Neither LM or HS had any involvement in CCMDD or clinical patient care. Focus groups were broadly organised by STREAM study arm, and by attendance in CCMDD. We audio recorded interviews and focus groups, and took accompanying field notes where appropriate. Recordings and notes were anonymised, transcribed and translated from isiZulu to English where necessary. Participants did not review transcripts or the analysis.

Data analysis and theoretical framework

We initially performed a deductive thematic analysis[13] using a framework based on predefined themes informed by theories of practice.[9] During analysis, we inductively identified new themes and codes that were derived from the data and integrated them into the pre-defined themes in the coding frame (Table S1). Data for this analysis was predominantly coded by JD, with assistance by LM, using NVivo 12.0 (QSR International, Melbourne, Australia).

Ethics

Ethical approval for this study was provided by the Biomedical Research Ethics Committee of the University of KwaZulu-Natal and the Institutional Review Board of the University of Washington.

Patient and public involvement

Patients and the public were not involved in the design, recruitment or conduct of the study, beyond being enrolled as participants. Results will be shared with the CAPRISA Community Advisory Board as part of dissemination activities.

RESULTS

Between March and August 2018 we undertook 25 interviews and four focus groups (five to ten participants) with a total of 55 clients, and eight interviews with healthcare workers. One client was both interviewed and also in a focus group. Amongst clients, median age was 31 years, 56.4% were female, 52.7% were employed and 58.2% had collected ART in CCMDD (Table 2). Amongst healthcare workers the median age was 39, 75.0% were women, and all were nurses

For peer review only - http://bmjopen.bmj.com/site/about/guidelines.xhtml

(Table 2). Interviews lasted 30-60 minutes and focus groups 60-80 minutes. Below, we present our findings, with supporting quotes, regarding the challenges to engagement in clinic-based services, and the influence of CCMDD on the social practice of engagement in HIV care. We use themes based on the four constructs of *materialities*, *competencies*, *meanings* and *other life practices* from theories of practice,[9] as well as the theme of problems with CCMDD implementation which we identified in the analysis (Table S1).

Table 2: Participants demographic and clinical characteristics

**Clients**			
		Frequency (N=55)	%
Age (years)	Median (IQR)	31 (27 - 37)	
Gender	Male	24	43.6
	Female	31	56.4
Ethnicity	Black African	55	100.0
Educational level	None or primary school	2	3.6
	Did not pass secondary school	14	25.5
	Passed secondary school	27	49.1
	Some tertiary education	12	21.8
Employed	Yes	29	52.7
	No	26	47.3
Income in ZAR per month (approximate USD)	<1000 (70)	23	41.8
	1000-4000 (70-280)	20	36.4
	> 4000 (280)	12	21.8
Has a regular/stable partner	Yes	42	76.4
	No	13	23.6
Number of children	None	10	18.1
	1 or more	45	81.8
Disclosed HIV status to anyone	Yes	52	94.6
	No	3	5.5
Travel time to clinic > 1 hour	Yes	7	12.7
	No	48	87.2
Distance travelled to clinic ≤ 5 kilometres	Yes	44	80.0
	No	11	20.0
Primary method of travel to clinic	Walking	3	5.5
	Public transport	49	89.1
	Private transport	3	5.5
VL <1000 copies/ml	Yes	49	10.9
	No	6	89.1
Collected ART in CCMDD	Yes	32	58.2

For peer review only

For peer review only - http://bmjopen.bmj.com/site/about/guidelines.xhtml

	No	23	41.8
**Healthcare Providers**			
Age (years)	Median (IQR)	39 (36 - 42)	
Gender	Male	2	25.0
	Female	6	75.0
Profession	Professional nurse	4	50.0
	Enrolled nurse	4	50.0

IQR, interquartile range

Materialities

Clients and healthcare workers described several ways in which the *materialities* of clinic-based HIV services presented challenges to ART provision, with overcrowded primary care clinics and long waiting times deterring people from collecting treatment.

> "*Sometimes I would end up not even collecting them [ART], because of the thought of the queue.*" Focus group 2, Participant 9, Female

> *"The clinic gets very full, you will wait there the whole day."* Focus group 3, Participant 3, Male

> *"When you are coming to the clinic, you must cancel all the plans you have and tell yourself that you will spend the rest of the day there. Because chances are you will finish late in the afternoon."* Focus group 2, Participant 1, Male

In contrast, many clients and healthcare workers described how CCMDD introduced changes in the *material circumstances* of ART delivery, with faster ART collection and more convenient opening hours.

> *"I think collecting at [the private pharmacy] is quicker than here. When I came back here at the clinic, I waited for a good two hours."* Focus group 1, Participant 7, Male

> *"They [patients] like CCMDD, [because] when they go to the pick-up points there's no queues like here in the clinic. They come in, they go straight to the pharmacies then they get their pack."* Staff interview 4

> *"Collecting… [at the community based organization] saves a lot of time, you don't spend 5 minutes. It doesn't get full, you can come anytime if it's still open. You just get in there, take your medication and leave."* Focus group 3, Participant 5, Female

For peer review only

These material differences between clinic-based ART provision and CCMDD, impacted and interacted with the other components of the social practice of engagement in HIV care.

Competencies

Clients described how time pressures and rushed appointments in clinics prevented communication with healthcare workers, which stopped them from gaining *competency* in understanding and managing their health and HIV treatment.

> *"They would just tell you your bloods are fine and you leave…We don't ask questions, we want to finish and allow other people to come in… there are a lot of patients [waiting] outside who also want to come in and be assisted.*" Client interview 3, Male

> *"Maybe you weren't feeling well two days ago and you want to tell the nurse about it, sometimes you can't… because you are looking at the situation the nurse is under. You see that she has no time for you and she will not tolerate you telling her other things."* Focus group 3, Participant 4, Female

Some clients also described being treated badly by clinic staff, further limiting their ability to become *competent* at taking medication.

> *"Sometimes you speak with [the nurse], and she gives you medication, [but] at the same time she's shouting at you. You can't do anything as a patient, you end up not knowing how you should take the medication."* Focus group 3, Participant 4, Female

Two healthcare workers expressed concerns that clients who become unwell in CCMDD could then have difficulty accessing clinical care. This could be overcome by communicating well to ensure that clients understood that they could return to the clinic at any time.

> "*What I don't like about CCMDD is that... you can say the patient is stable, but tomorrow is another story. So if such patients go to CCMDD… and something new develops… they [CCMDD staff] tell you that you must go back to your facility [by which time] … it will be late and the damage is done.*" Staff interview 7

> "*I don't think there's anything bad [about CCMDD] as such. But the thing is, if a patient becomes unstable, then there's nobody to care for them because at [the pickup point] they just issue the medication... the patient must be educated to return to us if they are not feeling well.*" Staff interview 5

For peer review only

Most clients seemed to understand that quicker ART collection through CCMDD came at the cost of receiving less clinical oversight , but understood that they could return to the clinic if they were unwell or needed additional care.

> *"It's quicker to just collect [at the community-based organisation], because you know how you are taking your treatment… because when all these things [measuring vital signs, seeing a nurse] are done, you will be delayed."* Focus group 2, Participant 8, Female

> *"…but at [the private pharmacy] you take your medication and leave, and it's good. When you have a problem, you come to the clinic."* Focus group 2, Participant 9, Female

However, one client refused referral into CCMDD because she liked having vital signs monitored and the opportunity to talk to nurses in clinic, while another client in CCMDD felt that it could be improved by a quick physical check-up in the community.

> *"They once said we can now get them [ART] outside... I don't want that, I am fine here [at the clinic]… You weigh, check blood pressure and then go to the nurse to collect medication and she will look at your results and tell you everything is going well, or you are lacking here and you are lacking here."* Client interview 16, Female

> *"I was relieved [that I was in CCMDD] because things were going to be quicker now, because this thing of staying all day at the clinic is not nice. But then the fact that you don't get checked up is not good. Otherwise, it's fine because it [the community-based organization] is quick. If your vital signs were done as well, it would have been better."* Client interview 5, Female

Clients not in CCMDD developed other *competencies* to overcome the multiple challenges to engagement in clinic-based care. These included sending relatives or friends to collect treatment, sharing ART, arriving at clinic very early in the morning to miss queues, and extreme patience because of the life-saving benefits and positive *meanings* associated with receiving ART.

> *"When it's my appointment date I wake up very early to get to the clinic… I will be here for the whole day… because there are a lot of people… You must wait that long and sometimes you get so tired and you wish you can just leave. What can we say? We must wait because it's our lives."* Client interview 12, Female

For peer review only

For peer review only - http://bmjopen.bmj.com/site/about/guidelines.xhtml

While successfully collecting ART in CCMDD required development of new *competencies*, some clients' previous experiences of buying from pharmacies was helpful.

> *"Ok, so [the first time I collected from the private pharmacy] I got in there and looked around to see where I'm supposed to go, because I'm used to just buying and then leaving. Ok, I saw that I need to go [to] the dispensary because I am coming to collect and I didn't have a problem I did everything well and I found a good person who was full of jokes."* Client interview 18, Female

Meanings

The physical organization of clinic services sometimes threatened clients' confidentiality, potentially revealing their HIV status, which for them *meant* potential exposure to HIV-related stigma.

> *"There are two polyclinics, there's an old one and the new one… [HIV] positive people go to the 'old poly', so when a person says, "He is an old poly" [it means] they saw you at the 'old poly'. They know when you go to the 'old poly' that you are coming for ART."* Focus group 1, Participant 1, Male

In order to manage the risk of HIV-related stigma in clinics, clients developed *competencies* to avoid disclosing their HIV status to people that they knew. Some reported pretending to collect treatment for someone else, or choosing to collect their treatment at a clinic far from home.

> "[There is] *another thing that makes us collect medication in other areas. We do have clinics in our local communities, [but] we are afraid to collect medication in those clinics because it is not safe… we are in one room with other patients and maybe we are only separated by a wall, so when I enter that door, people will see that I am going to collect my "qo" [ART]."* Focus group 3, Participant 7, Male

In contrast, several staff felt that CCMDD helped to change the *meanings* associated with HIV treatment by normalizing HIV services.

> *"Being on ART, it disrupted their [clients] normal lives, so with CCMDD people go back to their normal life routine. You don't have to feel that 'I am sick'… you don't have to be burdened with getting treatment.*" Staff interview 1

For peer review only - http://bmjopen.bmj.com/site/about/guidelines.xhtml

Specifically, collecting ART in private pharmacies, where clients could be getting non-HIV related medication, helped with maintaining confidentiality.

> *"[In the private pharmacy] they wrap the medication well and pack it well. They don't just give you un-wrapped medication where everyone can see. They wrap them and put them in a plastic bag…it looks like any medication you buy from [the pharmacy]."* Client interview 12, Female

However, as we found in clinics, stigma still affected the *meanings* of engagement with CCMDD. Some clients who collected from private pharmacies felt that they were treated differently, because they were not paying customers.

> *"They [the private pharmacy staff] were paying attention to the customers who were coming to buy. Because we don't come with money, we only come with our [CCMDD] cards, they weren't paying attention to us."* Client interview 8, Female

In some pharmacies there was a separate queue for CCMDD clients, which they felt could expose their HIV-positive status to others, despite people without HIV also collecting other chronic medication through CCMDD.

> *"Where I collect, there is our [CCMDD] queue and then there is a queue for everyone else. That is why I say sometimes they would say "these people are coming to collect their thing [ART]".* Focus group 1, Participant 1, Male

Furthermore, integrating HIV treatment with everyday life felt threatening to some clients. One declined referral to CCMDD because he was worried about revealing his HIV status and experiencing stigma.

> "Interviewer: *Okay, why don't you like going there [private pharmacy in CCMDD]?*
>
> Participant: *"Uhm… because this thing [collecting ART] is confidential. Imagine, since I'm working at the mall, how many people who live by my house come there? … Imagine a lady who stays by my place and sees me when I go to this. This is a secret... So, I would prefer to collect them here [at the clinic], because I don't want to feel embarrassed*." Client interview 3, Male

For peer review only - http://bmjopen.bmj.com/site/about/guidelines.xhtml

In general, clients understood that CCMDD was for stable patients, and associated being referred into CCMDD as *meaning* that they had been taking ART well. Some used this to motivate their own adherence.

> "*No one wants to go back to the clinic anymore, and I think that is why we are taking our medication so well... When the nurse showed me my results, I was relieved that I will be going back [to CCMDD]."* Focus group 2, Participant 9, Female

Other life practices

Many clients found that long waiting times in clinics and inflexible clinic opening hours (weekdays between 0700 to 1600 hours) clashed with employment opportunities and family responsibilities:

> "*Yes, but if I could be able to collect it on weekends… During the week I sometimes have temporary jobs somewhere, so I wish I could collect on weekends.*" Client interview 18, Female

> "*I did go to the clinic and the line was massive. So, I couldn't collect [ART], because I had to go back to work.*" Focus group 2, Participant 4, Male

> "*…because the time here [at clinic] clashes with my work time… When you come here [to the clinic] you do it for yourself and when you are at work you do it for the family, you see. You must choose sometimes.*" Client interview 24, Male

Quicker services in CCMDD and more flexible opening times meant that collecting ART was less disruptive for *other life practices*.

> "*Everything [at the community-based organisation] is perfect and fast. If… I'm supposed to start work at half past eight I can make it, because at eight o'clock I will be there and by ten past or quarter past I'm done.*" Client interview 14, Male

> "*[In CCMDD] the patients… are independent, they can go on a Saturday or after hours to pick up their medication, they don't have to take time off work to come to us [at the clinic].*" Staff interview 5

However, some pharmacies seemed to be placing restrictions on when ART could be collected, despite being open for other services. This inflexibility meant that some employed clients still had difficulties collecting their ART in CCMDD.

For peer review only

For peer review only - http://bmjopen.bmj.com/site/about/guidelines.xhtml

"The problem with [this private pharmacy] is that they don't issue treatment on weekends. So, since we are working, we don't have time because if I didn't come to work they will deduct a certain amount from my salary. When you go there in the afternoon, they tell you that they don't issue medication at the certain times. Which means I will not be getting my treatment whereas it is very important to me." Focus group 1 Participant 9, Female

Challenges with implementation of CCMDD Despite the general benefits of CCMDD, a few staff and clients also reported occasional problems with the implementation of the programme (Table 3). These included delays in receiving reminder SMS, the client's ART not being found at the pick-up point and inflexible pick-up dates at some pharmacies, with pickup points sending ART back to the central depot, if clients were a few days late (Table 3). At the time of the interviews, several pick-up points had reached capacity and were no longer accepting referrals, meaning that some clients had no pick-up points near to their homes. Staff also reported some barriers to registering people into CCMDD. Referral criteria require a valid identification card or passport, which excluded some clients. Furthermore, isoniazid prophylaxis therapy, a daily tablet to prevent tuberculosis, is not available in CCMDD, meaning clients could not be referred until they had completed the recommended twelve month prophylactic course. Lastly, the electronic prescription system for CCMDD was occasionally offline, which could make CCMDD registration more time consuming (Table 3).

Table 3. Challenges with implementation of CCMDD

Issue	Quotes
**Barriers to registering patients in CCMDD**	
Need for ID card	*"If a patient wants to be registered on CCMDD, strictly that patient must have a South African ID [or foreign passport] and every time the patient is going to collect the medication, they have to positively identify themselves using their ID. Something that we are not doing in the clinics. Some patients are not happy with that, ID's do get lost from time to time and then once the ID is not there, then they cannot give you that package at the pick-up point, then the patient would be forced to come back to the clinic… [and] then they get de-registered in the process."* Staff interview 4*
Problems with electronic prescription system	*"The online registration system crashes quite often so there's days where the nurses can't get online, they just have to register manually. It creates a problem because online registration is a lot quicker, so on the days when they have to do manual then they are backed up for quite a while, and then it just makes the work a lot more."* Staff interview 6
TB prophylaxis	*"There's a category of patients that can be stable on ARV's but still don't qualify for CCMDD. A typical point in example, if at any given*

For peer review only - http://bmjopen.bmj.com/site/about/guidelines.xhtml

	point you decide to put this patient on IPT, which is Isoniazid Preventive Therapy for TB, they don't take that patient. You see, for the patient to qualify for CCMDD they must be stable, but now CCMDD will not take a patient that is on IPT. So we have to keep that patient up until they finish the course of IPT before you can enrol them on CCMDD." Staff interview 4<br><br>*"they said because I am still taking the ones for protecting me against TB and I will finish them in August… after that I will hear from them [about referral to CCMDD]"* Client interview 21, Male.
**Problems with CCMDD implementation**	
SMS	*"I will receive the text message the following day in the afternoon, after I collected today. I never receive the text message [on time]. I receive it after."* Focus group 1 Participant 3, Female
ART not being found at the pick-up point	*"I once received an SMS and I went there [to the private pharmacy] the very same day I received the SMS, and I got there and they said my name is not there [on the list of people with ART to collect]. I then came here [to the clinic], and I got my treatment here. Fortunately, I had time on that day to end up coming here."* Focus group 2 Participant 4, Male
Inflexible pick-up dates	*The service providers are very strict when it comes to appointment dates, very strict. You get given a date and then you are expected to collect your parcel within 48 hours. After those two days then the medication will be sent back… and they get de-registered as a result. But here in the clinic they can miss the date by a day or 2 or 3 or 4 or 5, it's not that much of an issue.* Staff interview 4<br><br>*"It was my fault because I came after two days and I found that my treatment has been sent back [to the depot]."* Focus group 1 Participant 6, Male
Restricted ART pick-up times	*"They [private pharmacy staff] don't even take into consideration that I am coming from work… They are still at work and their job is to give me what I came here for [ART], but they tell me that they have closed and there is a queue."* Focus group 1, Participant 1, Male
Pick-up points reaching capacity	*"Most of the pick-up points were full, you know. They were full and some of the participants collect their medication far from where they stay, far from where they work you know. Jah, I think those were the disadvantages."* Staff interview 2

DISCUSSION

In this study, clients and healthcare workers identified several problems with collecting ART from clinics in Durban, South Africa. Using the themes of materialities, competencies, meanings and other life practices based on 'theories of practice',[9] we showed how these problems interact with, and impact on, engagement in HIV care. We present one of the first qualitative assessments of the largest differentiated ART delivery programme in the world and show how it overcame some of these problems, helped to normalise HIV treatment and reduced the impact

For peer review only - http://bmjopen.bmj.com/site/about/guidelines.xhtml

of collecting ART on other social practices in the clients lives. However, several challenges and inconsistencies remain in the implementation of CCMDD.

Materialities

We found several material barriers to engagement in clinic-based services, which are similar to findings from other studies. In a qualitative systematic review of 59 studies regarding long-term ART adherence and engagement in care in Africa, poor clinic services were identified as a major theme, with long waiting times, inflexible services and rigid and demanding policies all leading clients to disengage from care.[14] In our study, CCMDD changed some of the 'materialities' of ART provision, leading to clear practical benefits for clients. Similar findings have been reported in qualitative assessments of community adherence clubs in Mozambique, Kenya, Malawi, Zimbabwe and South Africa, which found reductions in clinic visits, waiting times, and transport costs for clients.[15–19]

Competencies

The material challenges to attending clinics interacted with other components of the social practice of engagement in HIV services. For example, in our study and in another study from South Africa, [20] rushed clinic appointments impeded communication with nurses, meaning there was no time for healthcare workers to equip patients with the competencies required to engage with HIV services and learn about taking ART. Accessing ART through the CCMDD programme also required clients to develop new skills and competencies. When the CCMDD system worked well, clients generally found it easy to navigate, and understood that they could return to their normal clinic if they needed additional care. However, a few healthcare workers expressed concerns that CCMDD could delay clients from accessing appropriate medical attention if they became unwell, and highlighted the need for adequate counselling of clients to ensure appropriate healthcare seeking. These findings are similar to a study of community adherence groups in Zimbabwe, where healthcare workers were worried about delayed access to care, while clients were happy to spend less time in clinics, and felt "empowered to visit the clinic whenever they needed".[19]

Meanings

We found that healthcare workers in clinics were often overburdened, sometimes disrespectful, and did not always maintain confidentiality, as has been seen in many other studies.[14] Clinics that separated HIV services from other care also threatened clients' confidentiality, therefore exposing clients to stigma, which has been widely reported as impacting negatively on

For peer review only - http://bmjopen.bmj.com/site/about/guidelines.xhtml

engagement in HIV care.[21,22] CCMDD also impacted on the meanings of engagement in HIV care. Attending private pharmacies was less burdensome, less obviously associated with being HIV positive, and more easily integrated with everyday life, thereby helping to normalise ART collection. In Kenya, integrated adherence clubs for non-communicable diseases and HIV were reported to reduce stigma, by normalising HIV to be like other chronic conditions.[16] However, we found multiple reports of preferential treatment by private pharmacy staff towards paying customers, suggesting some ongoing discrimination towards public sector CCMDD clients. Some clients also remained concerned that their HIV status could be revealed when collecting at community pick-up points, where they could be more likely to see neighbours or people they knew. In studies of community adherence clubs, clients were mainly concerned about revealing their HIV status to other group members, and therefore created strict rules around maintaining group confidentiality.[15–18] The risk of other adherence club members breaching confidentiality was offset by increased peer support from within the group.[15,17,19,20] In contrast, clients in CCMDD did not report benefits of peer support, but also did not have to negotiate group dynamics when collecting ART, as they tended to collect individually at pick-up points. Lastly, referral into CCMDD provided a new positive meaning to ART collection, and was seen as a reward and incentive to take ART well. Similarly, adherence clubs clients felt like staff were trusting them to manage their own HIV, and saw being sent back to the clinic as a punishment or failure to adhere to treatment.[20]

For peer review only

Other life practices

Several studies have described how the time spent travelling to clinics and waiting for ART can impact on clients' ability to seek or attend employment, and meet family responsibilities.[15,17,19] We found that a major benefit of CCMDD was reduced disruption to employment, although some working clients encountered problems with restricted collection times in a few private pharmacies. Similarly, in differentiated care programs in Malawi, Kenya and Zimbabwe, clients had more time to attend their businesses and farming,[17,19] and avoided taking days off work.[16,19]

Challenges with CCMDD implementation

As with other differentiated ART delivery programmes,[15–18] there were several reports of implementation problems that clients had to negotiate, such as delayed SMS reminders, ART not being available at the pickup point, and a few pharmacies placing restrictions on ART pick-up times. Furthermore, some of the eligibility criteria for CCMDD seemed to contradict other

For peer review only - http://bmjopen.bmj.com/site/about/guidelines.xhtml

stated aims of the healthcare system. For example, the need for an identification card could exclude the most vulnerable clients, while not providing tuberculosis prophylaxis within CCMDD could hamper tuberculosis control measures.[23]

Strengths and limitations

This is one of the first qualitative studies to assess differentiated ART delivery within the CCMDD programme. Our analysis is strengthened by the use of 'theories of practice' which highlighted the relationship between the materialities of ART provision, and how changes in these impacted on engagement in HIV care as well as clients' broader lives. Our interviewers were not involved in CCMDD or clinical patient care, which reduces the potential for social desirability bias. Limitations of our study include the use of a single urban clinic, and the use of clinical trial participants, meaning our findings may not be generalisable to other settings, such as rural areas. While our purposive sampling aimed to include a range of clients, clients who felt more positive towards CCMDD may have been more likely to participate. Lastly, the CCMDD programme is adapting quickly, and some of the early implementation problems that we report (e.g. pick-up points reaching capacity) may now have been resolved.

Implications

Our findings support the ongoing rollout of CCMDD, while highlighting areas where improvements are needed. Better regulation of private pharmacies could ensure consistent implementation of less restrictive ART collection times, and allow development of strategies to better protect confidentiality and prevent CCMDD clients from receiving inferior care. Increasing the flexibility of the ART collection date window could also increase clients' autonomy to collect on a day that suits them in CCMDD. Steps to reduce HIV-related stigma at a broader societal level are needed, as fear of disclosure remained a barrier to engagement in both clinic-based services and CCMDD. Further research is needed to establish the impact of CCMDD on clinical outcomes such as viral suppression and retention in care, and whether it is cost-effective. Of note, cost-effectiveness studies should attempt to capture the wider non-healthcare system costs and benefits for employed people, given that CCMDD may be particularly beneficial to them. Lastly, we also identify several areas that could be improved in clinic-based HIV services, including less restrictive opening times, more respectful treatment of clients, and better confidentiality.

For peer review only - http://bmjopen.bmj.com/site/about/guidelines.xhtml

Conclusion

In conclusion, we highlight how the material changes to ART provision in CCMDD facilitated engagement with HIV services, reduced disruption in clients' everyday lives, and helped to normalise HIV care. Improvements to the implementation of CCMDD could allow even greater benefits, and contribute towards achieving universal ART through more client-centred healthcare services.

COMPETING INTERESTS

The authors have no competing interests to declare.

ACKNOWLEDGEMENTS

We would like to thank Farzana Osman for descriptive analysis of participants' demographics. The support of the DST-NRF Centre of Excellence for HIV Prevention towards this research is hereby acknowledged. Opinions expressed, and conclusions arrived at, are those of the authors and are not necessarily to be attributed to the DST-NRF Centre of Excellence for HIV Prevention

FUNDING STATEMENT

The STREAM study was funded by the US National Institute for Health (AI124719-01). JD is funded by the Wellcome Trust PhD Programme for Primary Care Clinicians (216421/Z/19/Z). LM was funded by DST-NRF Centre of Excellence for HIV Prevention (UID96354). AG is supported by the South African Medical Research Council. STC was supported by funding from the National Institute for Health Research (NIHR) Health Protection Research Unit in Healthcare Associated Infections and Antimicrobial Resistance at University of Oxford in partnership with Public Health England (HPRU-2012-10041).

AUTHORS' CONTRIBUTIONS

JD, AG, HN, PKD and NG conceived and designed the study. LM and HS conducted interviews and focus group discussions, and transcribed and translated the data. JD, NG, HN and AG supervised data collection. JD and LM coded and analysed the data. AG, STC, GH and CB supervised data analysis. JD drafted the manuscript. JD, LM, AG, HS, STC, GH, CB, HN, PKD and NG critically reviewed and edited the manuscript and consented to publication.

For peer review only

For peer review only - http://bmjopen.bmj.com/site/about/guidelines.xhtml

DATA AVAILABILITY STATEMENT

No data are available

REFERENCES

1 Joint United Nations Programme on HIV/AIDS (UNAIDS). Communities at the Centre: Global AIDS Update 2019. Geneva, Switzerland: 2019. https://www.unaids.org/en/20190716_GR2019_communities

2 Grimsrud A, Barnabas R V., Ehrenkranz P, *et al.* Evidence for scale up: the differentiated care research agenda. *J Int AIDS Soc* 2017;**20**:22024. doi:10.7448/IAS.20.5.22024

3 Grimsrud A, Bygrave H, Doherty M, *et al.* Reimagining HIV service delivery: the role of differentiated care from prevention to suppression. *J Int AIDS Soc* 2016;**19**:21484. doi:10.7448/IAS.19.1.21484

4 Ehrenkranz P, Grimsrud A, Rabkin M. Differentiated service delivery. *Curr Opin HIV AIDS* 2018;**14**:1. doi:10.1097/COH.0000000000000509

5 Health Systems Trust. CCMDD Get Checked Go Collect: Free Chronic Medication. 2019.https://getcheckedgocollect.org.za/ccmdd (accessed 11 Feb 2020).

6 South African National Department of Health. Adherence Guidelines for HIV, TB and NCDs. Pretoria: 2016.

7 Roberts P. Centralized Chronic Medicine Dispensing and Distribution: A Public/Private Partnership to Increase Access to HIV/Chronic Medication. 2018. http://files.icap.columbia.edu/files/uploads/ICAP_Grand_Rounds_Project_Last_Mile_Slides.pdf

8 Meyer JC, Schellack N, Stokes J, *et al.* Ongoing initiatives to improve the quality and efficiency of medicine use within the public healthcare system in South Africa; A preliminary study. *Front Pharmacol* 2017;**8**:1–16. doi:10.3389/fphar.2017.00751

9 Skovdal M, Wringe A, Seeley J, *et al.* Using theories of practice to understand HIV-positive persons varied engagement with HIV services: a qualitative study in six Sub-Saharan African countries. *Sex Transm Infect* 2017;**93**:e052977. doi:10.1136/sextrans-2016-052977

10 Dorward J, Garrett N, Quame-Amaglo J, *et al.* Protocol for a randomised controlled implementation trial of point-of-care viral load testing and task shifting: the Simplifying HIV TREAtment and Monitoring (STREAM) study. *BMJ Open* 2017;**7**:e017507. doi:10.1136/bmjopen-2017-017507

For peer review only - http://bmjopen.bmj.com/site/about/guidelines.xhtml

11 Drain PK, Dorward J, Violette L, *et al.* Point-of-care HIV viral load testing combined with task shifting improves treatment outcomes: an open-label randomized controlled trial to Simplify HIV TREAtment and Monitoring (STREAM Study). *Lancet HIV* 2020;**epub ahead**.

12 Msimango L, Gibbs A, Shozi H, *et al.* Acceptability of point-of-care viral load testing to facilitate differentiated HIV care: a qualitative study of clients and healthcare workers. In: *International AIDS Society*. Mexico City, Mexico: 2019.

13 Attride-Stirling J. Thematic networks: an analytic tool for qualitative research. *Qual Res* 2001;**1**:385–405. doi:https://doi.org/10.1177/146879410100100307

14 Eshun-Wilson I, Rohwer A, Hendricks L, *et al.* Being HIV positive and staying on antiretroviral therapy in Africa: A qualitative systematic review and theoretical model. *PLoS One* 2019;**14**:e0210408. doi:10.1371/journal.pone.0210408

15 Rasschaert F, Telfer B, Lessitala F, *et al.* A qualitative assessment of a community antiretroviral therapy group model in Tete, Mozambique. *PLoS One* 2014;**9**:1–11. doi:10.1371/journal.pone.0091544

16 Venables E, Edwards JK, Baert S, *et al.* 'They just come, pick and go.' the acceptability of integrated medication adherence clubs for HIV and Non Communicable Disease (NCD) Patients in Kibera, Kenya. *PLoS One* 2016;**11**:1–12. doi:10.1371/journal.pone.0164634

17 Pellecchia U, Baert S, Nundwe S, *et al.* "We are part of a family". Benefits and limitations of community ART groups (CAGs) in Thyolo, Malawi: a qualitative study. *J Int AIDS Soc* 2017;**20**:21374. doi:10.7448/IAS.20.1.21374

18 Prust ML, Banda CK, Nyirenda R, *et al.* Multi-month prescriptions, fast-track refills, and community ART groups: results from a process evaluation in Malawi on using differentiated models of care to achieve national HIV treatment goals. *J Int AIDS Soc* 2017;**20**:21650. doi:10.7448/IAS.20.5.21650

19 Bochner AF, Meacham E, Mhungu N, *et al.* The rollout of Community ART Refill Groups in Zimbabwe: a qualitative evaluation. *J Int AIDS Soc* 2019;**22**. doi:10.1002/jia2.25393

20 Venables E, Towriss C, Rini Z, *et al.* Patient experiences of ART adherence clubs in Khayelitsha and Gugulethu, Cape Town, South Africa: A qualitative study. *PLoS One* 2019;**14**:e0218340. doi:10.1371/journal.pone.0218340

21 Turan B, Hatcher AM, Weiser SD, *et al.* Framing Mechanisms Linking HIV-Related Stigma, Adherence to Treatment, and Health Outcomes. *Am J Public Health* 2017;**107**:863–9. doi:10.2105/AJPH.2017.303744

22 Horter S, Bernays S, Thabede Z, *et al.* "I don't want them to know": how stigma creates

For peer review only

For peer review only - http://bmjopen.bmj.com/site/about/guidelines.xhtml

dilemmas for engagement with Treat-all HIV care for people living with HIV in Eswatini. *African J AIDS Res* 2019;**18**:27–37. doi:10.2989/16085906.2018.1552163

23 Tram KH, Mwangwa F, Chamie G, *et al.* Predictors of isoniazid preventive therapy completion among HIV-infected patients receiving differentiated and non-differentiated HIV care in rural Uganda. *AIDS Care - Psychol Socio-Medical Asp AIDS/HIV* 2019;**0121**. doi:10.1080/09540121.2019.1619661

FIGURE 1: SUMMARY OF THEORIES OF PRACTICE FRAMEWORK, REPRODUCED WITH PERMISSION FROM SKOVDAL ET AL., 2017[7]

For peer review only

For peer review only - http://bmjopen.bmj.com/site/about/guidelines.xhtml

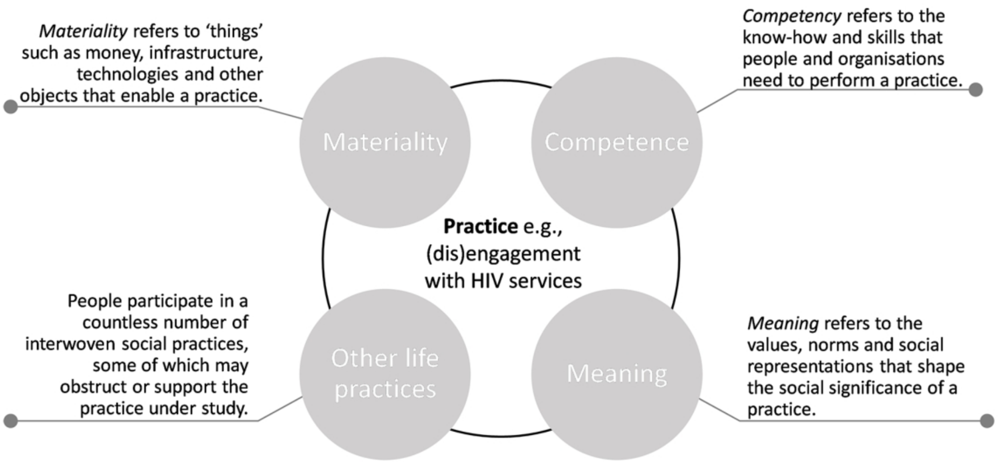

Summary of Theories of Practice Framework, reproduced with permission from Skovdal et al., 2017[7]

363x167mm (300 x 300 DPI)

Table S1: Coding frame based on Framework of Social Practice

Code	Description	Quote
**MATERIALITIES:** Physical 'things' that impact on engagement in HIV services		
**Characteristics of HIV services**	The physical aspects of HIV services that determine access	
Waiting times	Descriptions of waiting times in HIV services	*"I think collecting at [the private pharmacy] is quicker than here, when I came back here at the clinic I waited for a good two hours."* Focus group 1, Participant 7, Male<br><br>*"They [patients] like CCMDD, [because] when they go to the pick-up points there's no queues like here in the clinic. They come in, they go straight to the pharmacies then they get their pack."* Staff interview 4*
Numbers of patients	Descriptions of the number of patients receiving treatment	*"They [nurses] are always in a rush and there are a lot of us. Maybe we are more than 100 and there's a queue."* Focus group 1, Participant 4, Female<br><br>Interviewer: *"Ok, so the first time you went to the hall [CCMDD community pick-up point], how was it?"* Participant: *"It was nice because there was not a lot of us, I would just take my ID out and then take my treatment and leave."* Focus group 2, Participant 2, Male
Staffing levels	Descriptions of staffing levels in HIV services	*"There is not enough staff [in the clinic], everyone is rushing to finish... Maybe only three nurses are working, and they are seeing all these people. You are also rushing and she is also rushing and you are thinking as a patient that you left a lot of people outside [waiting to be seen after you]. So adding more staff would help."* Client interview 3, Male

For peer review only - http://bmjopen.bmj.com/site/about/guidelines.xhtml

Code	Description	Quote
Flexibility of HIV services	Descriptions of (in)flexibility of HIV services with regard opening hours and ability to change appointment days	*"Collecting on weekends would be a great idea, because you will not have an excuse especially if you are not working. Even at work they will have a problem if you have a specific day where you don't come to work or leave early [to collect ART]."* Focus group 2, Participant 1, Male<br>*"Collecting… [in CCMDD] saves a lot of time, you don't spend 5 minutes. It doesn't get full, you can come anytime if it's still open. You just get in there take your medication and leave."* Focus group 3, Participant 5, Female
Accessing other medical care apart from ART	Descriptions of how the ability to access other, non-ART related care influenced engagement with HIV services	*"It's quicker to just collect [ART] because you know how you are taking your treatment… When all these things [measuring vitals] are done you will be delayed."* Focus group 2, Participant 8, Female<br>*"The problem with it [CCMDD] is that you only come to collect your medication and then leave, they don't do other things as well. Like checking your blood pressure and other things to know how your health is."* Client interview 5, Female
Maintaining confidentiality	Descriptions of how the design of the service impacts on confidentiality	*"But at the nearby clinics, most of the nurses just display everything [ART] in front of everyone… there is no privacy in these clinics."* Focus group 1, Participant 8, Female<br>*"When your neighbours see you sitting there they will start to ask you questions. Even when they don't know why you are there, but they will know at the end why."* Focus group 1, Participant 6, Male
ART not being available in facility or pickup point	Descriptions of attending services but ART not being available	*"Sometimes you find that there is no treatment [in the clinic] and people would be scheduled for another date when the treatment is available."* Client interview 21, Male

Code	Description	Quote
Distance to HIV services	Descriptions of the distance and travel to HIV services affecting engagement in care	*"There are people who live far [from the clinic]… you find that they don't even have money for transport."* Client interview 6, Female
Barriers to engagement with CCMDD	Descriptions of factors that stop people from being referred into CCMDD or continuing to collect there (see Table 3)	See Table 3 for specific quotes
**Poverty**	The dynamics of lacking financial and material resources (e.g. food, other medication to manage side effects) that would support engagement with HIV services.	*"Fetching medication is problematic. You find that you don't have money, you need to fetch your medication and you don't have it [money]."* Client interview 1, Female
**COMPETENCIES:** Knowledge and skills that impact on engagement in HIV services		
**Communication between healthcare workers and patients**	The role of healthcare workers in communicating competencies and also clinical information about HIV and treatment to patients	*"Patient education… should be constant, it should be regular, regular talking with the patients, teaching patients how to live positively, considering things like the use of condoms, proper dieting and… the function of adherence."* Staff interview 4<br><br>Interviewer: *"When you ask the nurse about your results, what do they say?"*<br>Participant: *"You even get afraid to ask, because they don't have time, they are always rushing to see the next person."* Focus group 3, Participant 4, Female
Healthcare worker attitude	Descriptions of the importance of healthcare worker attitude in imparting these competencies towards patients	*"They [healthcare workers] must not judge us, they must do their job and what they were employed to do. Also, they need to learn to communicate, nurses can't communicate with people. You make one small mistake [and] they shout at you and talk to you the way they like."* Client interview 19, Female

For peer review only - http://bmjopen.bmj.com/site/about/guidelines.xhtml

Code	Description	Quote
**To manage stigma**	Knowledge and skills needed to manage the (non)disclosure of one's HIV status to others, and to deal with stigma from others including partners, family members and the wider community.	*"I didn't want to go to the clinic by where I live because I was afraid of people's eyes. Because when you start this [ART] people look at you in different way."* Client interview 20, Male
**To negotiate problems with HIV services**	Descriptions of strategies and skills that people have developed to mitigate problems with HIV services	*"You see, I make it a point that when it's my appointment date I wake up very early to get to the clinic… I will be here for the whole day… because there are a lot of people… You must wait that long and sometimes you get so tired and you wish you can just leave. What can we say? We must wait because it's our lives."* Client interview 12, Female<br><br>*"If you don't have a day-off on that day you can send someone to collect the medication for you."* Client interview 1, Female
**Social support**	The use of family and or the wider community as a source of knowledge to help maintain engagement with HIV services	"*[After starting ART] I did have a bad dream on the first day... But after that there were no problems. I asked other people, and they told me that they did have side-effects on the first day, you will have bad dreams and I didn't have a problem with that, it passed. Because when I asked other people they said, yes, it happens. But the ARVs are no longer the same as the previous ones, these ones are better because you only take one pill at night. Yes, I continued I took the medication."* Client interview 1, Female
**Maintaining a healthy lifestyle**	Knowledge and skills needed to maintain recommended lifestyle changes for people living with HIV	*"They were advising us that we shouldn't tell ourselves that we are going to die you see… that if you are person living with HIV you practice safe sex and not sleep around and try to eat healthy things in life. If you smoke reduce a bit and [reduce] drinking. General things were being spoken that we need to deal with."* Client interview 10, Male

For peer review only - http://bmjopen.bmj.com/site/about/guidelines.xhtml

Code	Description	Quote
**MEANINGS:** The meanings that are attached to HIV and engagement with HIV services		
**Stigma**	Prejudice and discrimination experienced by people living with HIV. Can be internal ('self-stigma') or external (enacted by others) and both perceived or actual.	*"[if there is a separate queue for ART] it's easier to be discriminated against, because they know what you are here for, so it's better for a person not to know what you are queuing for."* Focus group 1, Participant 4, Female<br><br>*"I wish they could make this disease [HIV] like flu, and not something scary. That is the reason why people don't even want to come close to you."* Focus group 1, Participant 7, Male<br><br>*"My concern is the stigma we have as infected people, we get treated as people who don't live in this world. So, we try to hide ourselves."* Focus group 2, Participant 4, Male
**Normalisation of HIV**	The portrayal of HIV as a common and normal experience share with many others, that does not have to affect health if ART is taken.	Interviewer: *"When you found out [that you were HIV-positive] … how did you feel about that?"*<br>Participant: *"I didn't feel like a different person, I didn't see any difference, I saw myself as a normal person who needs to take care of herself and take her medication properly so life can go on. I didn't feel any change and I wasn't sick at all."* Client interview 5, Female
**Meanings of CCMDD**	Descriptions of the meaning associated with being referred to CCMDD including reasons for being referred	*"They transferred me to CCMDD because I was taking my treatment properly and I also know that I was taking my treatment well."* Focus group 2, Participant 7, Male
**Public and private treatment**	The meanings associated with receiving government funded versus private care	*"Sometimes you wait for other [customers] to buy and they [private pharmacy staff] will serve the other people before you. Even if you are in the queue, it's like they are sifting us [public CCMDD clients] from the crowd."* Focus group 1, Participant 5, Female

For peer review only - http://bmjopen.bmj.com/site/about/guidelines.xhtml

Code	Description	Quote
		"They [private pharmacy staff] were paying attention to the customers who were coming to buy. Because we don't come with money, we only come with our [CCMDD] cards and ID's [so] they were not paying attention to us." Client interview 8, Female
**Experience of HIV and treatment**	Having had personal experience, or having witnessed in others, the effects of HIV and life-saving treatment. Having faith in treatment and HIV services in restoring health and life	*"As for me, even when I walk on the streets I feel healthy and confident because I know I am taking my medication properly."* Focus group 2, Participant 9, Female
**OTHER LIFE PRACTICES:** Other repeated activities of daily life that clients engage in that impact on engagement in HIV services		
**Employment**	The duties and daily activities of employment and formal and informal work	*"I even go after work, I knock off at 4 and go there [CCMDD pick-up point] and take my medication. Even during my break time, I go, or when I am swamped I ask [my supervisor] and quickly rush there take my medication and come back… What I love [about CCMDD] is that I don't need to take a day off at work to collect medication at the clinic and wait for the whole day. Now at work I ask for that 20 minutes and go there [to CCMDD]. It's fine. I don't skip work, and there is no one that sees me."* Client interview 12, Female<br><br>*"I think it [CCMDD] is good, because clinics get full. It's better because if you work you can quickly go there [the community pick-up point], collect and come back. I think it's a good method."* Client interview 4, Female
**Family life**	Activities of daily life that revolve around family	*"Last time [I had to collect ART from the clinic] you know what happened? I had to send my wife. I'd got a part time job, so I couldn't manage to come. [So I asked her], 'Please just go with my card to collect my medication'. She doesn't want to come back here [to the clinic] ever again.*

Code	Description	Quote
		Because of the time that she spent [waiting for treatment].” Client interview 14, Male

*To protect participant confidentiality, we have not provided complete demographic details of healthcare worker for individual quotes.

For peer review only

For peer review only - http://bmjopen.bmj.com/site/about/guidelines.xhtml

Understanding how community antiretroviral delivery programmes influence engagement in HIV care: a qualitative assessment of the Centralised Chronic Medication Dispensing and Distribution programme in South Africa

Supporting Information

Table of Contents **Page**

1. Participant in-depth interview topic guide 1

2. Staff in-depth interview topic guide 3

3. Participant focus group topic guide – CCMDD 5

For peer review only

For peer review only

1

Patient and service provider perspectives regarding point of care viral load testing and differentiated care: an ancillary study to the STREAM study

Participant in Depth Interview Topic Guide

The overarching objectives of this interview topic guide are to:

- Understand participants experience of laboratory and point of care viral load testing
- Understand participants experience of receiving care from an enrolled nurse compared to a professional nurse
- Understand participants experience of receiving HIV care at clinics, community based organisations and pharmacies.

The interview should last approximately 30 to 60 minutes. The questions are indicative of the topics covered and will be shaped by participants' discussions and concerns. The interview will be conducted by a skilled Zulu facilitator and recorded without participant's name or personal details, but with their consent and permission for further analysis.

Introduction and background experience of HIV care

Tell me a little about yourself, your age, where you stay, what you do to get by, and where you have been getting your treatment from.

Laboratory and point of care testing

Tell me about the last time you had a blood test as part of your HIV care. When did you get the results?

How did you feel while you were waiting for the results?

Tell me about the results that you were given. How did you feel about these results?

If you had point of care blood taken, what were the good things and what were the bad things about this approach?

What would you want us to do differently with blood testing? Why do you think this would help?

Antiretroviral therapy collection at community centres and pharmacies

Tell me about the last time that you collected your antiretrovirals (ARVs). Where did you go, who did you talk to and how was the experience?

What were the good things about collecting treatment there, and what were the bad things? If you ever collected treatment at a community centre or community pharmacy, can you tell me the story of the first time you went there?

What other things are going on in your life which make it easier or harder to collect your treatment?

Enrolled nurse and professional nurse care

Can you tell me about a time when you felt unwell and told your problem to a nurse? What happened? How did you feel about the care you received?

Tell me about the last time you came to clinic and saw a nurse. What did the nurse do for you that day?

For peer review only - http://bmjopen.bmj.com/site/about/guidelines.xhtml

If you saw an enrolled nurse, rather than a professional nurse, how did you feel about the care you received from the enrolled nurse?

What were the good and bad things about seeing an enrolled nurse?

Wrap Up

Have you got any other ideas that may help improve HIV care services?

For peer review only

For peer review only - http://bmjopen.bmj.com/site/about/guidelines.xhtml

Patient and service provider perspectives regarding point of care viral load testing and differentiated care: an ancillary study to the STREAM study

Staff in Depth Interview Topic Guide

The overarching objectives of this interview are to:

- Understand staff experience and views on laboratory and point of care viral load testing
- Understand staff experience and views regarding care provision by an enrolled nurse compared to a professional nurse
- Understand staff experience and views regarding care provision at clinics, community based organisations and pharmacies.

The interview should last approximately 30 to 60 minutes. The questions are indicative of the topics covered and will be shaped by the discussions and interviewees concerns. The interview will be conducted by a skilled facilitator and recorded without participant's name or personal details, but with their consent and permission for further analysis.

Introduction and background experience of HIV care

Tell me a little about yourself, where you have worked in the past, where you work now and what your main role at work is.

Laboratory and point of care testing

Tell me about any point of care (POC) tests that are used in your clinic/area of work, and your experience of using them.

What do you like and not like about each of these POC tests?

How could these POC tests be improved?

In your experience, what are the most important features of POC tests?

Tell me about the last time you told a patient that they had a high viral load. If this was a POC test, do you think giving the result on the day made any difference? What difference would there have been if it was a laboratory test?

If this was a laboratory test, what difference would there have been if it was a POC test?

Tell me the ways that you think POC testing could improve HIV care?

ART collection at community centres and pharmacies (CCMDD)

Tell me about your experience of the Centralised Chronic Medicines Dispensing and Distribution (CCMDD) system. What are the good things and bad things about it?

Tell me the main reasons that patients like CCMDD.

Tell me the main reasons that patients don't like CCMDD.

How do you think the CCMDD system could be improved?

For peer review only - http://bmjopen.bmj.com/site/about/guidelines.xhtml

Enrolled nurse and professional nurse care

What do you feel are the most important roles that enrolled nurses currently perform in the HIV programme?

What do you feel are the most important roles that professional nurses currently perform in the HIV programme?

Do you think any professional nurse roles could be done by enrolled nurses? Which ones and why/why not?

Are there any roles that professional nurses DON'T currently perform that you think they could or should do?

In our study, enrolled nurses have been seeing stable patients. What advantages and disadvantages do you think this approach has?

Are there any ways that you think we could improve this approach?

Wrap Up

Have you got any other ideas of how POC testing, community ART delivery/CCMDD or using enrolled nurses in innovative ways may help improve HIV care services?

For peer review only

For peer review only - http://bmjopen.bmj.com/site/about/guidelines.xhtml

For peer review only

Patient and service provider perspectives regarding point of care viral load testing and differentiated care: an ancillary study to the STREAM study

Participant Focus Group Topic Guide - CCMDD

We will arrange to meet participants enrolled in the study in a private location at or near the clinic to participate in a focus group. Between 5-10 participants will be in each focus-group.

The overarching objectives of this focus group topic guide are to:

- Understand participants experience of receiving HIV care at clinics, community based organisations and pharmacies.

The focus groups should last approximately 45-75 minutes. The questions are indicative of the topics covered and will be shaped by participants' discussions and concerns. The group will be facilitated by a skilled Zulu facilitator and recorded without participants' names or personal details, but with their consent and permission for further analysis

Introduction and background experience of HIV care

Tell us a little about your lives, your age, where you stay, what you do to get by, and where you have been getting your treatment from.

ART collection at community centres and pharmacies

Can someone who had a problem getting their treatment in the last few months tell us the story about what happened?

Did anyone else in the group experience something similar and manage to find a way around it – can you describe this please?

Is there anything that the CAPRISA or CDC clinic could have done to help you overcome these problems?

Does anyone have examples of other strategies that you or other people use to fit collecting medication into your everyday life?

Some people have been able to collect their treatment from community pharmacies and community centres. Can someone who did this tell us about the first time they went to collect their treatment there, where did you go, who did you talk to and how was the experience?

Does anyone else have a similar story? What were the good things about collecting treatment there, and what were the bad things? How did you feel when staff first told you that you could collect treatment at the community centre or pharmacy? Can anyone tell us about what happens when they come back to the clinic for review every 6 months?

Wrap Up

Have any of you got any other ideas that may help improve HIV care services?

For peer review only - http://bmjopen.bmj.com/site/about/guidelines.xhtml

COREQ (COnsolidated criteria for REporting Qualitative research) Checklist

A checklist of items that should be included in reports of qualitative research. You must report the page number in your manuscript where you consider each of the items listed in this checklist. If you have not included this information, either revise your manuscript accordingly before submitting or note N/A.

Topic	Item No.	Guide Questions/Description	Reported on Page No.
**Domain 1: Research team and reflexivity**			
Personal characteristics			
Interviewer/facilitator	1	Which author/s conducted the interview or focus group?	
Credentials	2	What were the researcher's credentials? E.g. PhD, MD	
Occupation	3	What was their occupation at the time of the study?	
Gender	4	Was the researcher male or female?	
Experience and training	5	What experience or training did the researcher have?	
Relationship with participants			
Relationship established	6	Was a relationship established prior to study commencement?	
Participant knowledge of the interviewer	7	What did the participants know about the researcher? e.g. personal goals, reasons for doing the research	
Interviewer characteristics	8	What characteristics were reported about the inter viewer/facilitator? e.g. Bias, assumptions, reasons and interests in the research topic	
**Domain 2: Study design**			
Theoretical framework			
Methodological orientation and Theory	9	What methodological orientation was stated to underpin the study? e.g. grounded theory, discourse analysis, ethnography, phenomenology, content analysis	
Participant selection			
Sampling	10	How were participants selected? e.g. purposive, convenience, consecutive, snowball	
Method of approach	11	How were participants approached? e.g. face-to-face, telephone, mail, email	
Sample size	12	How many participants were in the study?	
Non-participation	13	How many people refused to participate or dropped out? Reasons?	
Setting			
Setting of data collection	14	Where was the data collected? e.g. home, clinic, workplace	
Presence of non-participants	15	Was anyone else present besides the participants and researchers?	
Description of sample	16	What are the important characteristics of the sample? e.g. demographic data, date	
Data collection			
Interview guide	17	Were questions, prompts, guides provided by the authors? Was it pilot tested?	
Repeat interviews	18	Were repeat inter views carried out? If yes, how many?	
Audio/visual recording	19	Did the research use audio or visual recording to collect the data?	
Field notes	20	Were field notes made during and/or after the inter view or focus group?	
Duration	21	What was the duration of the inter views or focus group?	
Data saturation	22	Was data saturation discussed?	
Transcripts returned	23	Were transcripts returned to participants for comment and/or	

For peer review only

Topic	Item No.	Guide Questions/Description	Reported on Page No.
		correction?	
**Domain 3: analysis and findings**			
Data analysis			
Number of data coders	24	How many data coders coded the data?	
Description of the coding tree	25	Did authors provide a description of the coding tree?	
Derivation of themes	26	Were themes identified in advance or derived from the data?	
Software	27	What software, if applicable, was used to manage the data?	
Participant checking	28	Did participants provide feedback on the findings?	
Reporting			
Quotations presented	29	Were participant quotations presented to illustrate the themes/findings? Was each quotation identified? e.g. participant number	
Data and findings consistent	30	Was there consistency between the data presented and the findings?	
Clarity of major themes	31	Were major themes clearly presented in the findings?	
Clarity of minor themes	32	Is there a description of diverse cases or discussion of minor themes?	

Developed from: Tong A, Sainsbury P, Craig J. Consolidated criteria for reporting qualitative research (COREQ): a 32-item checklist for interviews and focus groups. *International Journal for Quality in Health Care*. 2007. Volume 19, Number 6: pp. 349 – 357

Once you have completed this checklist, please save a copy and upload it as part of your submission. DO NOT include this checklist as part of the main manuscript document. It must be uploaded as a separate file.

For peer review only
